# Lymphatic vessels in bone support regeneration after injury

**DOI:** 10.1016/j.cell.2022.12.031

**Published:** 2023-01-19

**Authors:** Lincoln Biswas, Junyu Chen, Jessica De Angelis, Amit Singh, Charlotte Owen-Woods, Zhangfan Ding, Joan Mane Pujol, Naveen Kumar, Fanxin Zeng, Saravana K. Ramasamy, Anjali P. Kusumbe

**Affiliations:** 1 Tissue and Tumor Microenvironments Group, MRC Human Immunology Unit, MRC Weatherall Institute of Molecular Medicine, Medical Sciences Division, University of Oxford, Oxford OX3 9DS, UK; 2 MRC London Institute of Medical Sciences, Imperial College London, London W12 0NN, UK; 3 Heidelberg University Biochemistry Center, Im Neuenheimer Feld 328, Heidelberg D-69120, Germany; 4 State Key Laboratory of Oral Diseases, West China Hospital of Stomatology, Sichuan University, Chengdu 610041, China; 5 Department of Clinic Medical Center, Dazhou Central Hospital, Dazhou, China

**Keywords:** lymphatic vessels, bone, regeneration, lymphangiocrine, 3D imaging, aging, injury, hematopoiesis, stress, IL6

## Abstract

Blood and lymphatic vessels form a versatile transport network and provide inductive signals to regulate tissue-specific functions. Blood vessels in bone regulate osteogenesis and hematopoiesis, but current dogma suggests that bone lacks lymphatic vessels. Here, by combining high-resolution light-sheet imaging and cell-specific mouse genetics, we demonstrate presence of lymphatic vessels in mouse and human bones. We find that lymphatic vessels in bone expand during genotoxic stress. VEGF-C/VEGFR-3 signaling and genotoxic stress-induced IL6 drive lymphangiogenesis in bones. During lymphangiogenesis, secretion of CXCL12 from proliferating lymphatic endothelial cells is critical for hematopoietic and bone regeneration. Moreover, lymphangiocrine CXCL12 triggers expansion of mature Myh11^+^ CXCR4^+^ pericytes, which differentiate into bone cells and contribute to bone and hematopoietic regeneration. In aged animals, such expansion of lymphatic vessels and Myh11-positive cells in response to genotoxic stress is impaired. These data suggest lymphangiogenesis as a therapeutic avenue to stimulate hematopoietic and bone regeneration.

## Highlights

• Light-sheet imaging of intact bones uncovers lymphatic vessels in bones
• Stress-induced IL6 drives lymphatic vessel expansion
• Lymphatic vessels mediate bone and hematopoietic regeneration
• Lymphangiocrine CXCL12 supports regeneration in bone

## Graphical abstract

**Figure:**
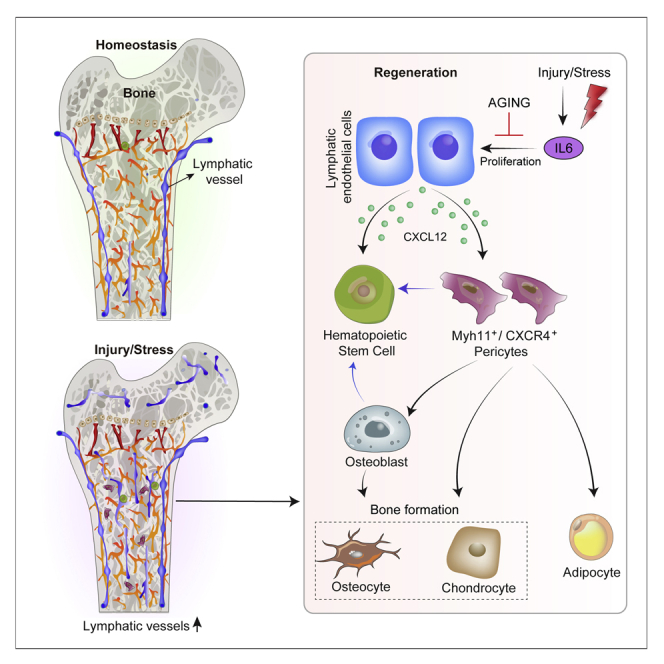

## Teaser

Lymphatic vessels not only connect secondary lymphoid organs with tissue sites throughout the body but are also now shown directly irrigating bones and participating in their regeneration.

## Introduction

Vasculature, a key component of the bone marrow microenvironment, provides signals for the maintenance and proliferation of hematopoietic stem and progenitor cells in bone.^1^^,^^2^^,^^3^^,^^4^ Vasculature also regulates the differentiation of perivascular mesenchymal stem cells to generate bone cells.^5^^,^^6^^,^^7^^,^^8^^,^^9^ However, due to technical difficulties with imaging calcified tissues, the organization of blood vessels in bone has, until recently, remained elusive. Advancements in imaging thick slices of murine bones have now provided insights into the heterogeneity of blood vessels in bone.^10^^,^^11^^,^^12^ Together with functional studies, this work demonstrated that the skeleton and the bone marrow endothelium form a functional unit that is important during development, homeostasis, and aging.^12^^,^^13^^,^^14^ Specifically, a specialized capillary termed “type H” regulates both angiogenesis and osteogenesis and couples the two processes within the bone.^12^ Blood vessels also provide distinct niches for the maintenance and proliferation of hematopoietic stem cells (HSCs).^1^^,^^2^

The lymphatic system regulates fluid homeostasis, waste clearance, and immune responses.^15^^,^^16^^,^^17^ Until recently, it was believed that certain tissues, such as the brain, eye, and bone, lack lymphatics. However, recent work has revealed the presence of lymphatic vessels in the dura mater of the mouse brain and the spinal vertebral column.^18^^,^^19^^,^^20^ Further, the eye tissue hitherto believed to lack lymphatic circulation harbors the Schlemm’s canal, which is analogous to lymphatics.^21^^,^^22^ Nevertheless, the current dogma is that the bone and bone marrow lack lymphatic vessels and that lymphatic vessel growth in the bone may be detrimental, as seen in Gorham-Stout disease, a rare bone disorder characterized by the improper growth of lymphatic vessels in bones.^23^^,^^24^ Some historic studies have pointed to a different conclusion. For example, earlier investigations indicated that Indian ink injected into long bones reaches the lymph nodes.^25^^,^^26^ Moreover, when injected into the bone marrow, high-molecular-weight molecules such as ferritin and horseradish peroxidase are able to reach the periosteal surface of the bone,^27^^,^^28^^,^^29^^,^^30^ suggesting a path connecting the two regions.

The identification of lymphatic endothelial cell (LEC) markers, such as lymphatic vessel endothelial hyaluronan receptor 1 (LYVE1), prospero-related homeobox 1 (PROX1), and podoplanin, has accelerated the characterization of lymphatic vessels in several organs over the last decade.^31^^,^^32^^,^^33^^,^^34^^,^^35^^,^^36^^,^^37^ However, a study using LYVE1 and podoplanin failed to identify lymphatic vessels in 2D analysis of thin human bone sections.^30^ Immunolabeling and 3D imaging of intact skeletal tissues is technically challenging due to their calcified nature. The current methods for clearing and immunolabeling whole and intact bones are limited^25^^,^^26^^,^^38^^,^^39^^,^^40^ and time consuming or generate low-resolution data.

Here, to more definitively investigate whether lymphatic vessels exist within bones, we devised a method to immunolabel and image intact bones at high resolution on a light-sheet microscopy platform. We applied this light-sheet imaging and functional genetics to bone tissues. We found that lymphatic vessels do exist in bone and drive bone and hematopoietic regeneration.

## Results

### Light-sheet imaging of intact skeletal elements reveals lymphatic vessels in bones

**Figure 1:**
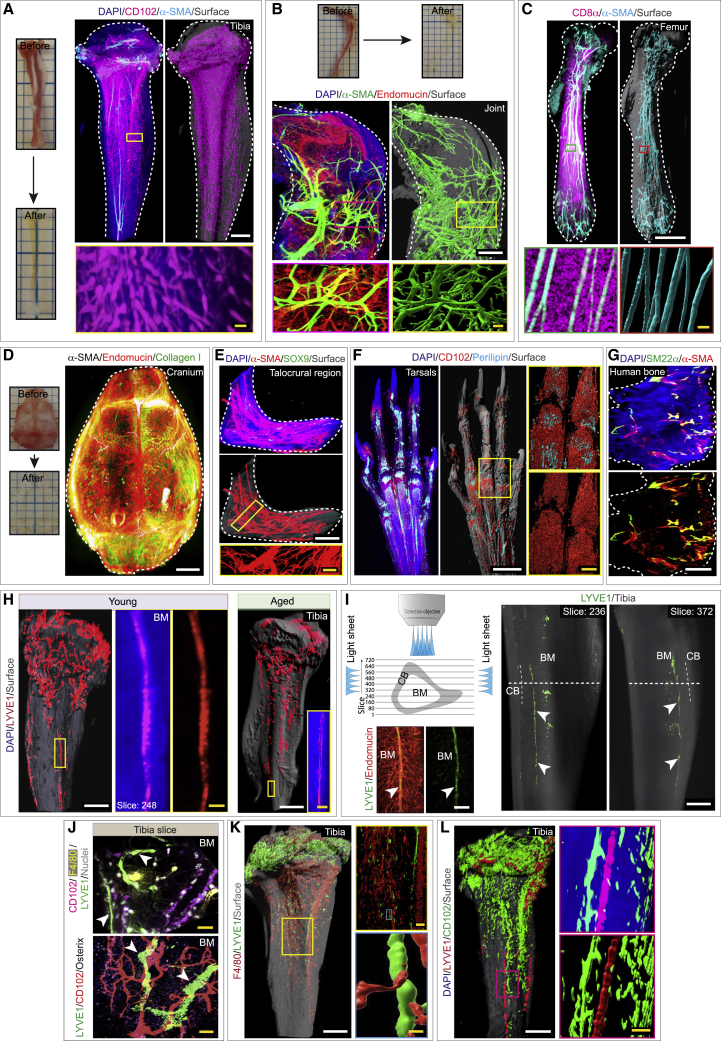
Light-sheet imaging of intact skeletal elements identifies lymphatic vessels in whole bones (A) Images of tibiae prior to and post-tissue clearing. 3D images were acquired on a light-sheet microscopy platform post-clearing with a high-magnification inset; labeled with the nuclear stain DAPI, immunostained with CD102 and α-SMA. (B) Murine knee joint prior to and after tissue clearing. 3D images of the knee joint; labeled with DAPI, α-SMA, and endomucin. (C) Murine femur immunostained with CD8α and α-SMA. (D) Murine cranium prior to and post-tissue clearing; immunolabeled with α-SMA, endomucin, and collagen I. (E) 3D images of the talocrural region with DAPI, α-SMA, and SOX9. (F) Images of the murine tarsal bones with DAPI, CD102, and perilipin. (G) Representative 3D images of human bone biopsy with DAPI, SM22α, and α-SMA. (H) 3D images of tibiae from young and aged mice with DAPI and LYVE1. (I) Schematic showing the image acquisition strategy on a light-sheet microscopy platform. 3D images of a tibia with LYVE1 and endomucin. Arrowheads, lymphatic vessels. (J) 3D image showing high-magnification inset of a murine tibia labeled with CD102, F4/80, and LYVE1. 3D image showing immunostaining for LYVE1, CD102, and osterix. Arrowheads, lymphatic vessels. (K) Representative 3D images of a murine tibia with F4/80 and LYVE1. (L) Image of a whole tibia showing DAPI, LYVE1, and CD102. Bone marrow, BM; cortical bone, CB. Scale bars: white, 500 μm; yellow, 50 μm. See also Figures S1, S2, and S3 and Videos S1 and S2.

The current immunolabeling methods for intact skeletal elements are limited and time consuming and generate low-resolution images. We modified the existing methods and developed a pipeline for efficient clearing and immunolabeling of intact skeletal tissues that enable rapid single-cell resolution and quantitative panoptic 3D light-sheet imaging (Figures 1A–1F; Video S1). The method enables ultrafast immunolabeling and clearing of calcified tissues within 3.5 days and will accelerate discoveries compared to other imaging pipelines.

Video S1. Panoptic multicolor immunolabeling and imaging of diverse calcified tissues, related to Figure 1

**Figure S1:**
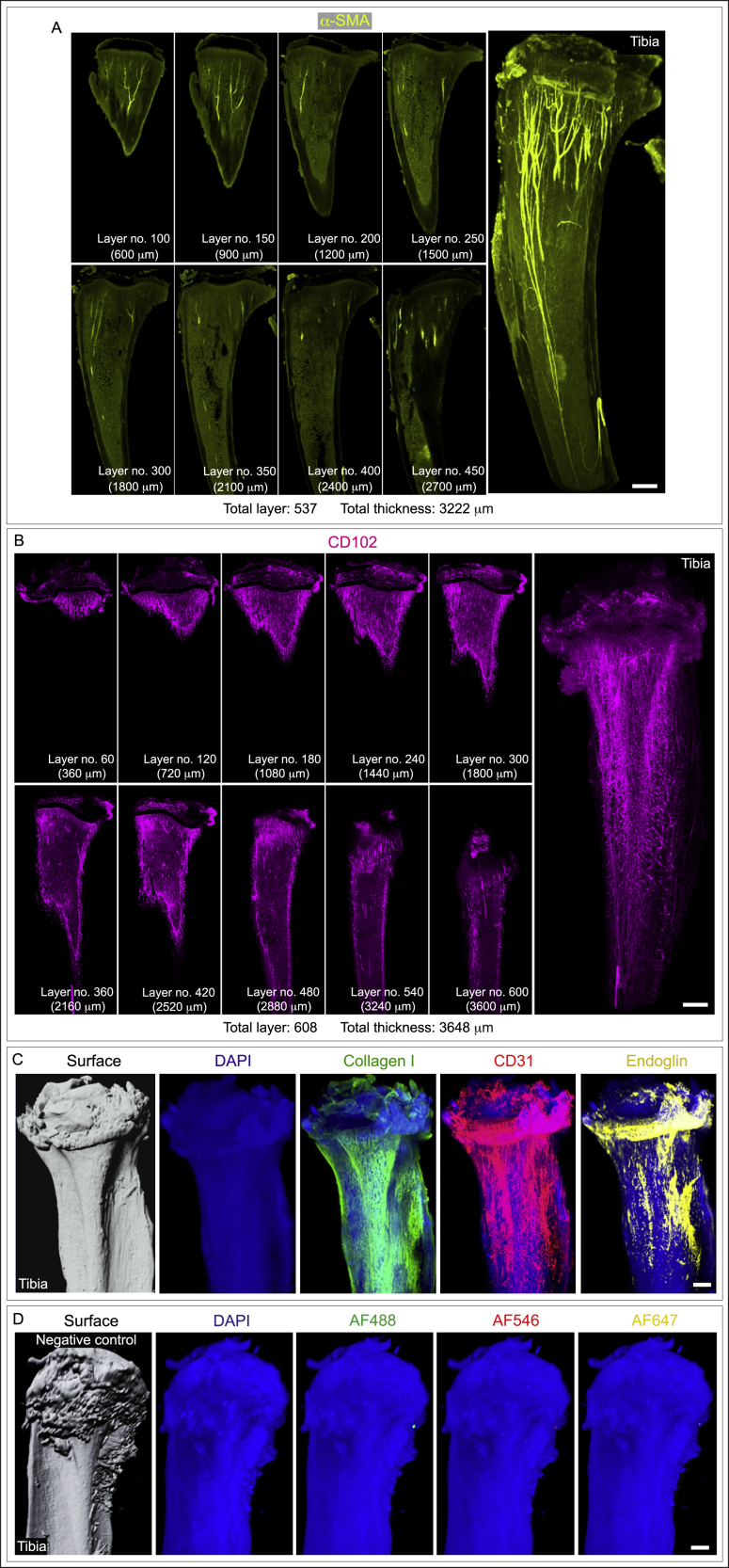
3D and slice view images showing penetration of antibodies throughout the tissue and negative controls used during immunostaining for light-sheet imaging, related to Figure 1 (A) 3D image of a whole murine cleared tibia acquired on a light sheet microscopy platform showing α-SMA immunostaining (right panel). Images (left panels) display α-SMA immunostaining and antibody penetration throughout multiple layers in this tibia which consists of 537 layers with a total thickness of 3222 μm. Scale bar: 500 μm. (B) 3D image of a whole murine cleared tibia acquired on a light sheet microscopy platform showing CD102 immunostaining (right panel). Images (left panels) display CD102 immunostaining and antibody penetration throughout multiple layers through this tibia which consists of 608 layers and a thickness of 3648 μm. Scale bar: 500 μm. (C and D) Representative 3D images of whole murine tibial bones acquired on a light sheet microscope. Immunostaining was performed with primary antibodies for Collagen I (green), CD31 (red), and Endoglin (yellow) and Alexa fluor conjugated secondary antibodies - Alexa Fluor 488 (AF488: green), Alexa Fluor 546 (AF546: red) and Alexa Fluor 647 (AF647: yellow) in positive controls (C). In negative controls (D) primary antibodies not added and stained only with secondary antibodies - Alexa Fluor 488 (AF488: green), Alexa Fluor 546 (AF546: red), and Alexa Fluor 647 (AF647: yellow). Scale bars: 500 μm.

**Figure S2:**
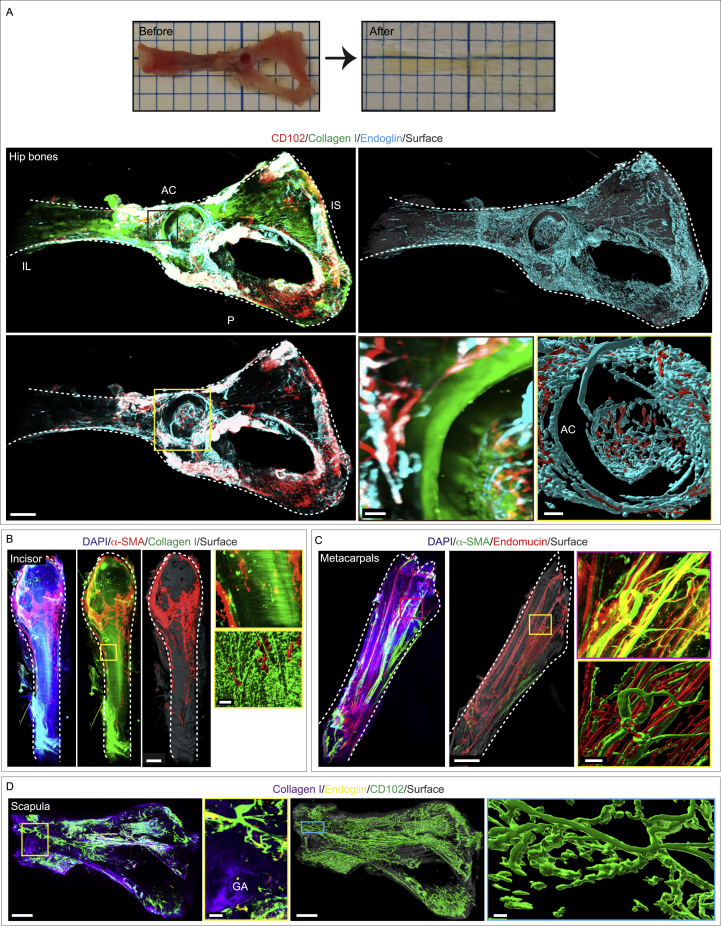
Immunostaining, clearing and multicolor panoptic light-sheet imaging of different intact murine bones, related to Figure 1 (A) Images of murine hip bones prior to (top-left panel) and post (top-right panel) tissue clearing. 3D images of the hip bones (center-left panels) with high magnification insets of the AC region (bottom-right panels); immunostained with CD102 (red), Collagen I (green) and Endoglin (cyan) (ilium: IL; ischium: IS; pubis: P and acetabulum: AC) with a surface overlay of the hip bone (gray). Scale bars: 800 μm and insets 150 μm (brown) and 200 μm (yellow). (B) 3D images of a murine incisor (left panels) with high magnification insets (right panels); labeled with DAPI, α-SMA, and Collagen I. Scale bars: 300 μm and insets 80 μm. (C) 3D images of murine metacarpal bones (left panels) with high magnification insets (right panels). Immunostaining for α-SMA (green) and Endomucin (red). Scale bars: 800 μm and insets 150 μm. (D) 3D image of the scapula with high magnification insets of the glenoid cavity (center-left and right panels: GA, glenoid cavity); immunolabeled with Collagen I, Endoglin and CD102. Scale bars: 500 μm and insets 80 μm.

The addition of collagenase digestion step after fixation and decalcification allows rapid and through antibody penetration. In line with this, immunostained bones showed antibody penetration and staining deep throughout the stained bones and calcified tissues (Figures S1A and S1B), while the negative controls without primary antibodies lacked staining (Figures S1C and S1D). Such an approach allowed visualization of the bone marrow microenvironment at a high resolution and in 3D across diverse murine whole bones and hard tissues (Figures 1A–1F and S2A–S2D) and human bone biopsies (Figure 1G).

**Figure S3:**
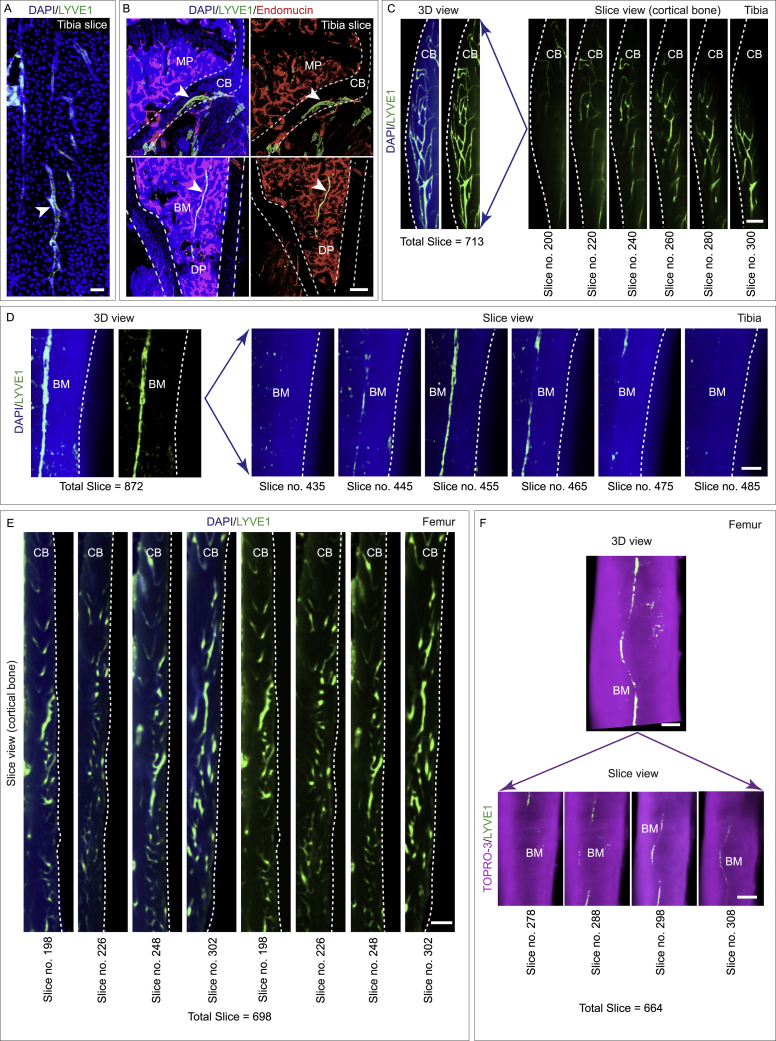
3D imaging showing localization of lymphatic vessels in healthy murine bones, related to Figures 1 and 2 (A) 3D confocal image of a murine tibial bone labeled with DAPI and LYVE1. Arrowhead denotes the lymphatic vessels. Scale bar: 50 μm. (B) 3D images of murine tibial bones; labeled with DAPI, LYVE1 and Endomucin (metaphysis: MP; diaphysis: DP; cortical bone: CB; bone marrow: BM). Arrowheads: lymphatic vessels. Scale bar: 400 μm. (C) Representative 3D images with LYVE1 immunostaining of a whole murine tibial bone (total slice number, 713) showing the cortical region. Images in slice view show LYVE1 immunostaining across the cortical region of this whole bone. Note the distribution of LYVE1^+^ lymphatic vessels through the cortical region of the bone. Cortical bone: CB. Scale bar: 300 μm. (D) Representative 3D images of a whole murine tibial bone showing the marrow region (total slice number, 872). Images in slice view show LYVE1 immunostaining across the layers of bone marrow. Bone marrow: BM. Scale bar: 300 μm. (E) Representative 3D images (with 698 slices) with LYVE1 immunostaining of a whole murine femur showing the cortical region. Images showing slices across this cortical region of this whole bone. Note the distribution of LYVE1^+^ lymphatic vessels through the cortical region of the bone. Cortical bone: CB. Scale bar: 400 μm. (F) Representative 3D images of a whole murine femur bone showing the marrow region (total slice number, 664). Images in slice view show LYVE1 immunostaining across the layers of bone marrow. TOPRO-3 showed in magenta. Bone marrow: BM. Scale bars: 300 μm.

**Figure 2:**
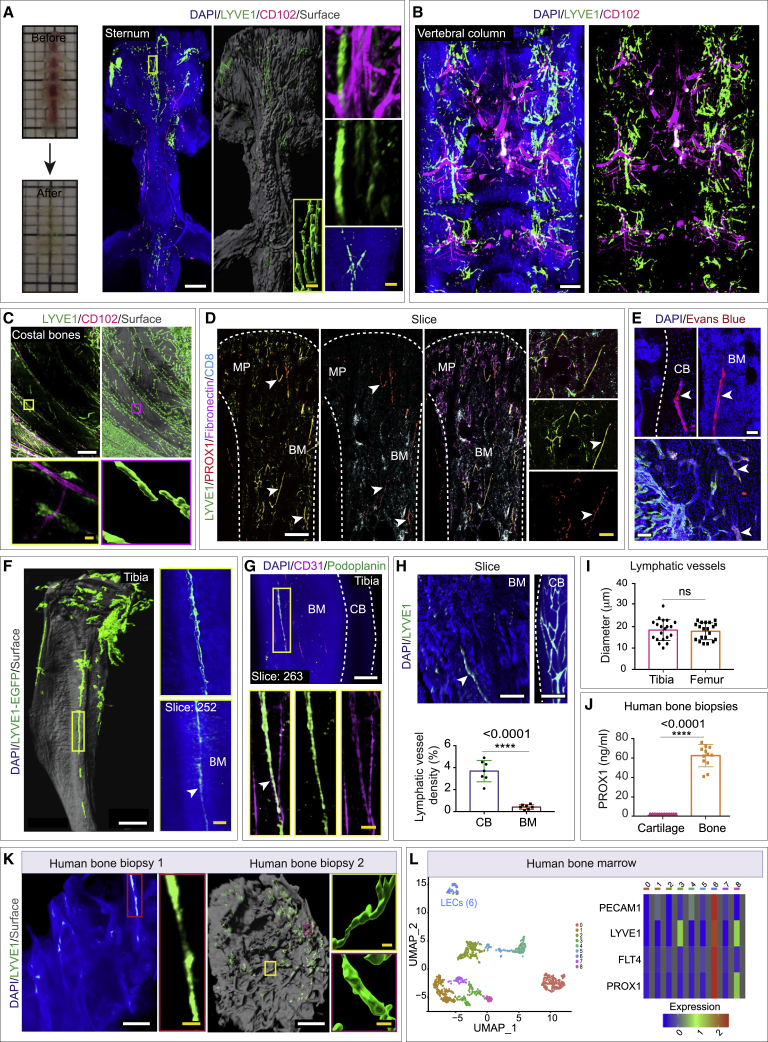
Distribution of lymphatic vessels in mouse and human bones (A) Images of a murine sternum prior to and post-tissue clearing and stained with DAPI, LYVE1, and CD102. (B) 3D images of a murine vertebral column labeled with DAPI, LYVE1, and CD102. (C) Costal bones with LYVE1 and CD102. (D) 3D images of a tibia showing immunostaining for LYVE1, PROX1, fibronectin, and CD8. Arrowheads indicate PROX1 and LYVE1 double-positive cells. (E) 3D images of tibiae from mice after the injection of Evans blue dye into the inner leg, medial to the tail, and footpad; DAPI and Evans blue dye. Arrowheads, Evans blue. (F) 3D image of a *Lyve1 EGFP Cre* adult (12 weeks old) mouse tibia; DAPI and LYVE1-EGFP. Arrowhead shows a lymphatic vessel. (G) Murine tibia with CD31, podoplanin, and DAPI labeling. Arrowhead, co-localization of podoplanin and CD31. (H) 3D images showing LYVE1-positive lymphatic vessels in BM versus CB. Arrowhead shows a lymphatic vessel. Quantification of lymphatic vessel density in CB and BM of murine tibiae (n = 7). (I) Quantification of lymphatic vessel diameter (n = 20). (J) Quantification of PROX1 concentration by ELISA (n = 12). (K) 3D images of human bone marrow biopsies from two donors with LYVE1 and DAPI. (L) UMAP projection of single cells from human bones showing different endothelial cell subsets, including the LECs (6). Heatmap showing PECAM1, LYVE1, FLT4, and PROX1 expressions in different endothelial cell subsets. Two-tailed unpaired t tests (^ns^p > 0.05 and ^∗∗∗∗^p < 0.0001). Scale bars: white, 500 μm; yellow, 50 μm. n represents biological replicates. Bone marrow, BM; metaphysis, MP; cortical bone, CB. See also Figures S3 and S4 and Video S2.

**Figure S4:**
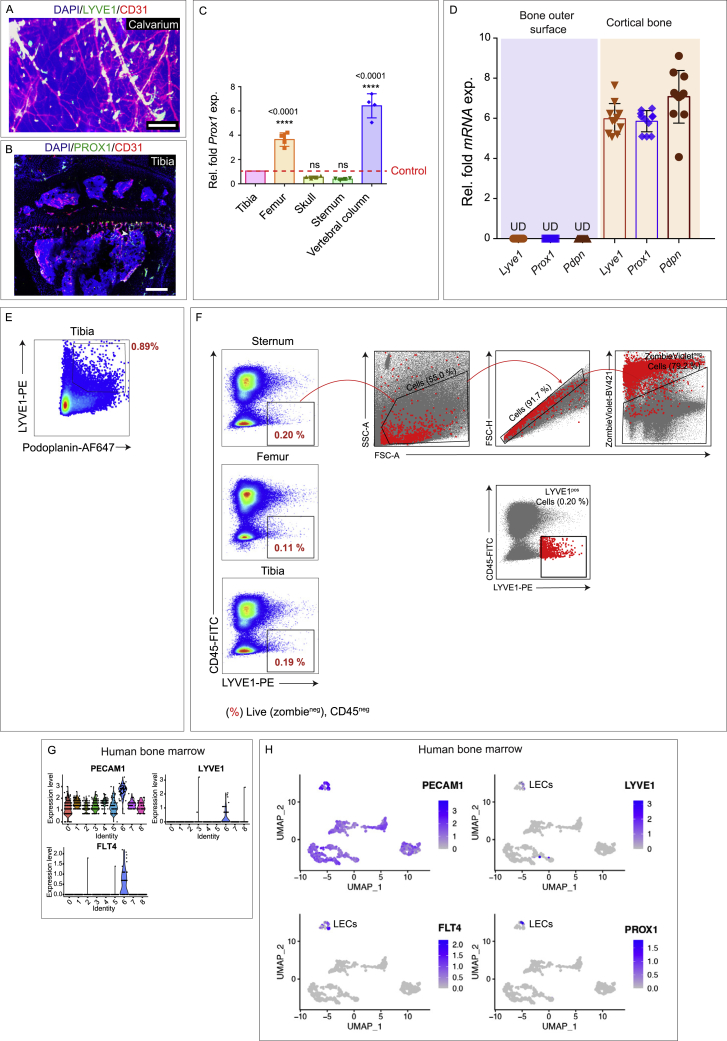
Analysis of lymphatic vessels and LECs in different bones, related to Figure 2 (A) 3D image of a murine calvarium showing DAPI (blue), LYVE1 (green), and CD31 (red). Scale bar: 600 μm. (B) 3D image of a murine tibial bone showing DAPI (blue), PROX1 (green) and CD31 (red). Arrowhead shows a lymphatic vessel. Scale bar: 300 μm. (C) Quantitative analysis of *Prox1 mRNA* expression in various bones - femur, skull, sternum and vertebral column as compared to tibia (mean ± SEM, *n*= 4 biological replicates). p values derived from two-tailed unpaired t-tests (^ns^p>0.05 and ^∗∗∗∗^p < 0.0001). (D) Relative fold *mRNA* expressions of *Lyve1*, *Prox1*, and *Pdpn* in mRNA isolated from the bones outer surface versus the isolated from the cortical region of the bones after processing for light sheet imaging (*n* = 11 biological replicates). Undetectable: UD. (E and F) Representative flow cytometry panels of lymphatic endothelial cells (LECs) isolated from various murine bones as gated (E) zombie-dye^-^, CD45^−^, Podoplanin^+^ and LYVE1^+^. (F) Zombie-dye^-^, CD45^**−**^, and LYVE1^+^ with back gating insets. (G) Violin Plots of single cells from human bone marrow presented the expression of PECAM1, LYVE1 and FLT4 genes in a single cluster. (H) UMAP projections showing LECs in the single cell suspension from human bone marrow.

To determine whether bones contained lymphatic vessels, we performed immunolabeling with several different lymphatic vessel markers. We observed LYVE1-positive lymphatic vessels on cleared murine whole bones (Figures 1H–1L, S3A, and S3B; Video S2), both in the cortical regions and the bone marrow cavity, but more abundant in the cortical regions (Figures S3C–S3F). Both whole-bone 3D rendering and single z-plane projections of the light-sheet images revealed LYVE1-positive lymphatic vessels (Figures 1H–1L, S3A, and S3B). Indeed, we observed LYVE1-positive lymphatic vessels across different murine bones, including sternum, vertebral column, costal bones, femur, calvarium, and hip bones (Figures 2A–2C, S3E, S3F, and S4A; Video S2). LYVE1, used together with CD102 also called ICAM2, a member of an intracellular adhesion molecule family, is expressed by blood vessels but not by lymphatic vessels (Figures 1J, 1L, and 2A–2C). Another lymphatic endothelial marker, PROX1, also displayed lymphatic vessels in bones (Figures 2D and S4B), and qPCR demonstrated *Prox1* expression across diverse murine bones (Figure S4C). qPCR analysis with *Lyve1*, *Prox1*, and *Pdpn* expressions in bones processed for light-sheet imaging did not detect *Lyve1*, *Prox1*, and *Pdpn* expressions in the outer surface of bone (Figure S4D). For further analysis and identification of lymphatic vessels in bone, we used Evans blue dye, a functional tool to map lymphatic vessels.^12^^,^^41^^,^^42^ Evans blue, a vital dye, binds with high-affinity tissue protein and is selectively and exclusively absorbed from interstitial space by initial lymphatic vessels.^12^^,^^43^^,^^44^^,^^45^ Evans blue was injected subcutaneously into the inner leg, medial to the tail and footpad.^12^^,^^41^^,^^43^^,^^46^^,^^47^ Imaging of tibial bones showed the uptake of Evans blue, and its accumulation in tibial bones confirmed the presence of lymphatic vessels (Figure 2E). In addition to immunostainings, light-sheet imaging of intact bones from the lymphatic vessel reporter mouse line LYVE1-EGFP^48^ also detected LYVE1-positive lymphatic vessels in bones (Figure 2F; Video S2). Finally, immunostaining with the LEC marker podoplanin^49^ detected lymphatic vessel structures in bones (Figure 2G).

Video S2. Multiple murine bones showing the presence of lymphatic vessels in bones, related to Figures 1 and 2

LECs were also detected in the single-cell suspension of bones using LYVE1 and podoplanin by flow cytometry. Since podoplanin is also expressed by a subset of mesenchymal cells and LYVE1 by a subset of macrophages, we identified double-positive LYVE1^+^ podoplanin^+^ cells (Figure S4E). In addition, we confirmed the presence of LECs in single-cell suspension of bones as CD45^−^ LYVE1^+^ cells, this confirmed the presence of LECs in different bones including sternum, femur, and tibia (Figure S4F). Furthermore, immunostaining with macrophage marker, F4/80, in intact bones confirmed that the LYVE1-positive vascular structures are negative for F4/80 (Figures 1J and 1K). The density of lymphatic vessels in cortical bones was higher compared to in the bone marrow, and the diameter of these vessels was ∼19 μm (Figures 2H and 2I).

To determine whether these findings were relevant to the human context, we also examined the presence of lymphatic vessels in human bones by performing enzyme-linked immunosorbent assay (ELISA) for PROX1 and immunostaining with LYVE1 on human bone biopsies. PROX1 expression was detectable in all the human bone biopsies analyzed but was undetectable in primary cartilage cells cultured from these bone samples (Figure 2J). 3D imaging of LYVE1 immunostaining also showed lymphatic vessels in cleared human bones (Figure 2K). Further, analysis of our single-cell sequencing data of human bone marrow demonstrated the presence of a small fraction of LECs (Figures 2L, S4G, and S4H). Thus, our imaging data, combined with other analyses, establish the presence of lymphatic endothelial cells within bone tissue in both human and mice.

### Lymphatic vessels in bones expand during genotoxic stress

Our own and other studies previously indicated that blood vessels in bones are highly susceptible and severely affected during radiation and myeloablation.^6^^,^^50^^,^^51^^,^^52^ Specifically, the bone marrow sinusoidal endothelium dilates and type H endothelial cells expand, while the number of sinusoidal endothelial cells declines upon radiation injury.^11^

**Figure 3:**
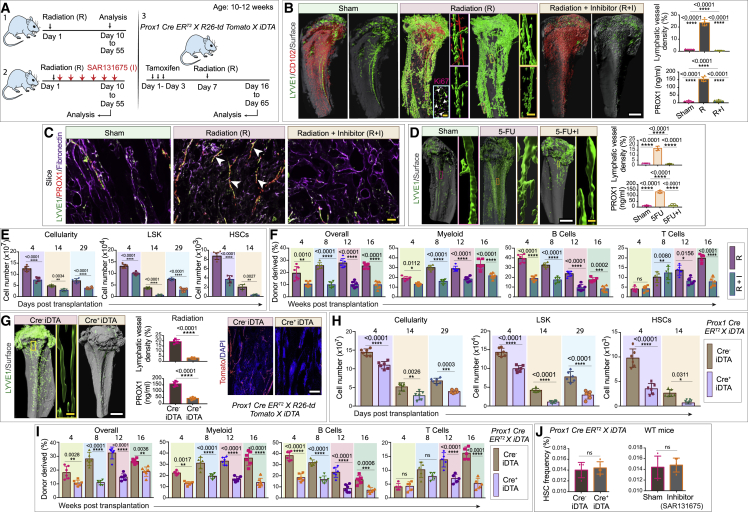
Lymphatic vessels in bones drive hematopoietic regeneration (A) Schematic depicting the experimental design, whereby wild-type or *Prox1 Cre ER*^*T2*^*X R26-td Tomato X iDTA* mice were subjected to radiation prior to bone collection. In some cases, mice were additionally treated with a VEGFR3 inhibitor (SAR131675: I) immediately after radiation (R) and for every successive 48 h up until 10 days. (B) 3D images of tibiae showing LYVE1, CD102, and Ki67. Mice were subjected to radiation (R) or radiation along with SAR131675 (I) inhibitor treatment (R + I) compared with the unirradiated PBS-injected (sham) mice. Arrowheads, Ki67^+^ LECs. Quantification of lymphatic vessel density (n = 5). ELISA quantification of PROX1 concentration from R, R + I, and unirradiated PBS-treated (sham) mice (n = 6). (C) 3D images showing immunolabeling for LYVE1, PROX1, and fibronectin. Mice were subjected to radiation (R) or radiation along with SAR131675 (I) inhibitor treatment (R + I) and unirradiated PBS-injected (sham). Arrowheads, PROX1 and LYVE1 double-positive cells. (D) 3D images of tibiae with LYVE1 from 5-fluorouracil (5-FU) or 5-FU along with SAR131675 (I) inhibitor (5-FU + I)-treated mice compared to PBS-injected (sham) mice. Quantification of lymphatic vessel density in these mice (n = 5). ELISA quantification of PROX1 concentration in tibiae from 5-FU, 5-FU + I, and sham mice (n = 6). (E) Bone marrow (BM) cellularity, number of LSK cells, and HSCs in bones from radiation (R) and radiation along with SAR131675 (I) inhibitor (R + I)-treated mice. Analysis was performed at 4, 14, and 29 days post-radiation and BM transplantation (n = 6). (F) 1 × 10^6^ donor BM cells from radiation (R) or radiation along with SAR131675 (I) inhibitor (R + I)-treated mice (as detailed in E) were transplanted into secondary wild-type recipient mice (n = 6). Overall, myeloid, B cells, and T cells reconstitution were assessed at 4, 8, 12, and 16 week post-radiation and BM transplantation. (G) 3D images showing LYVE1 or Tomato and DAPI from Cre^−^ iDTA and Cre^+^ iDTA; *Prox1 Cre ER*^*T2*^*X R26-td Tomato X iDTA* mice subjected to radiation. Quantification of lymphatic vessel density in bones from Cre^−^ iDTA and Cre^+^ iDTA mice subjected to radiation (n = 5). ELISA quantification of PROX1 concentration (n = 6). (H) Bone marrow cellularity, number of LSK cells, and HSCs in bones from Cre^−^ iDTA and Cre^+^ iDTA, Prox1 *Cre ER*^*T2*^*X iDTA* mice were analyzed at 4, 14, and 29 days post-radiation and BM transplantation (n = 6). (I) 1 × 10^6^ donor BM cells from Cre^−^ iDTA or Cre^+^ iDTA, *Prox1 Cre ER*^*T2*^*X iDTA* primary donor mice (as detailed in H) were transplanted into secondary wild-type recipient mice. Overall, myeloid, B cells, and T cells reconstitution were assessed at 4, 8, 12, and 16 week post-radiation and BM transplantation (n = 6 overall, myeloid, and B cells; n = 5 for T cells). (J) Frequency of HSCs in *Prox1 Cre ER*^*T2*^*X iDTA* bones from Cre^−^ iDTA and Cre^+^ iDTA mice (n = 5). Frequency of HSCs in SAR131675 inhibitor-treated wild-type mice compared to PBS-injected (sham) mice (n = 5). One-way ANOVA tests with Tukey’s multiple comparisons tests ( B and D); two-way ANOVA with Sidak multiple comparisons tests (E, F, H, and I); two-tailed unpaired t tests (G and J). ^ns^p > 0.05, ^∗^p < 0.05, ^∗∗^p < 0.01, ^∗∗∗^p < 0.001, and ^∗∗∗∗^p < 0.0001. Scale bars: white, 500 μm; yellow, 100 μm. n represents biological replicates. See also Figure S5.

**Figure S5:**
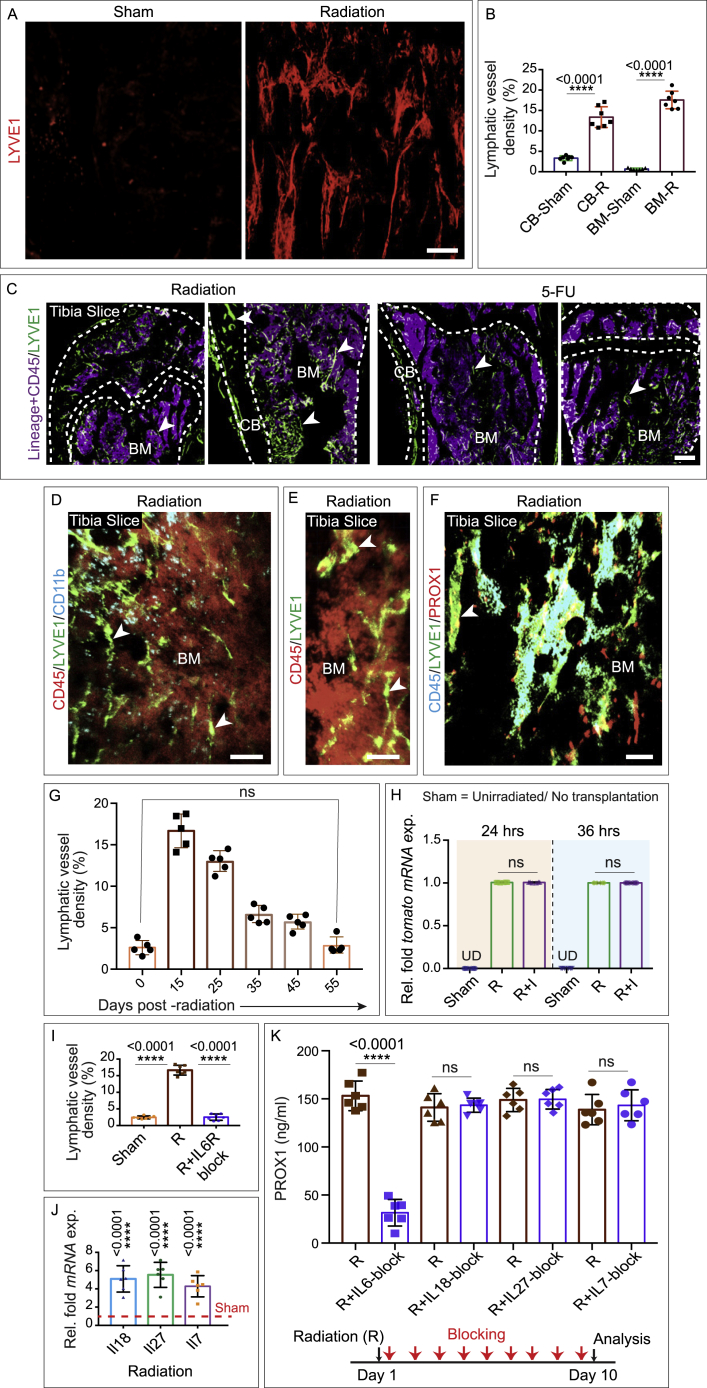
Analysis of lymphatic endothelium during genotoxic stress, related to Figures 3 and 4 (A) High magnification images demonstrating LYVE1 (red) staining of the tibia in healthy mice (sham) or mice subjected to radiation. Scale bar: 200 μm. (B) Quantification of lymphatic vessel density in tibial bones from mice treated with radiation compared to unirradiated sham (*n* = 7 biological replicates). Cortical bone: CB and bone marrow: BM. Statistical significance between each group was determined using two-tailed unpaired t-tests (^∗∗∗∗^p < 0.0001). (C) Representative 3D images of tibial bones from radiation and 5-FU treated mice showing CD45 (violet) and LYVE1 (green). Arrowheads denote the lymphatic vessels (cortical bone: CB and bone marrow: BM). Scale bar: 300 μm. (D) Confocal image of a murine tibia immunolabeled with CD45 (red), LYVE1 (green), and CD11b (cyan). Arrowheads denote the lymphatic vessels (bone marrow: BM). Scale bar: 200 μm. (E) Representative image of a murine tibia showing immunostaining for CD45 (red) and LYVE1 (green). Arrowheads indicate the lymphatic vessels (bone marrow: BM). Scale bar: 200 μm. (F) 3D image of a murine tibia showing immunostaining for CD45 (cyan), LYVE1 (green), and PROX1 (red). Arrowhead: lymphatic vessels (bone marrow: BM). Scale bar: 150 μm. (G) Quantification of lymphatic vessel density in tibial bones from mice were analyzed at 0-, 15-, 25-, 35-, 45-, and 55 days of post-radiation (*n* = 5 biological replicates). p values derived from two-tailed unpaired t-tests (^ns^p>0.05). (H) Relative fold *tomato mRNA* expression in tibial bones from unirradiated (sham), radiation (R) treated or radiation with SAR131675 (I) inhibitor treated (R + I) mice (*n* = 6 biological replicates). Undetectable; UD. p values derived from two-tailed unpaired t-tests (^ns^p>0.05). (I) Quantification of lymphatic vessel density in tibial bones from unirradiated (sham), radiation treated (R) and radiation along with IL6R blocking antibody (R + IL6R-block) treated mice, (*n* = 7 biological replicates). p values derived from one-way ANOVA tests with Tukey’s multiple comparisons tests (^∗∗∗∗^p < 0.0001). (J) Relative fold *mRNA* expression of *Il18*, *Il27* and *Il7* in tibial bones from radiation treated mice compared to unirradiated sham mice (*n* = 6 biological replicates). p values derived from two-tailed unpaired t-tests (^∗∗∗∗^p < 0.0001). (K) ELISA quantification of PROX1 concentration in tibial bones from radiation (R) only mice as compared to radiation along with blocking (block) antibodies for IL6 (R + IL6-block), IL18 (R + IL18-block), IL27 (R + IL27-block) and IL7 (R + IL7-block) (*n* = 6 biological replicates). Statistical significance between each group was determined using two-tailed unpaired t-tests (^ns^p>0.05 and ^∗∗∗∗^p < 0.0001).

To determine whether and how lymphatic vessels are affected, we analyzed the impact of radiation injury and myeloablation on the bone lymphatic endothelium (Figure 3A). To do so, we lethally irradiated mice, followed by transplantation of one million bone marrow cells. Bones from irradiated mice exhibited a dramatic expansion of lymphatic vessels. This was revealed by light-sheet imaging of LYVE1-positive lymphatic vessels and quantification of PROX1 by ELISA (Figures 3B, 3C, and S5A–S5F). Based on immunostaining by the proliferation marker Ki67, it seems likely that the observed post-radiation expansion of lymphatic vessels involves LEC proliferation (Figure 3B). Furthermore, analysis of lymphatic vessel density showed that vessel expansion in the bone peaked at 15 days post-radiation, subsequently returning to a normal density by 55 days post-radiation (Figure S5G).

Finally, to confirm the specificity of these observations, we examined mice treated with a lymphangiogenesis inhibitor. VEGFR3 is a known regulator of lymphangiogenesis that is selectively inhibited by SAR131675.^11^^,^^53^ When we treated irradiated mice with SAR131675, we no longer observed expansion of bone lymphatic vessels, as demonstrated via imaging of LYVE1-expressing lymphatic vessels and ELISA quantification of PROX1 concentration (Figures 3A–3C). Analogous to radiation, chemotherapy-driven genotoxic stress induced by 5-fluorouracil (5-FU) treatment also led to the expansion of lymphatic vessels in bones as demonstrated by imaging of LYVE1-expressing vessels and quantification of PROX1 concentration by ELISA (Figures 3D and S5C). Further, when we treated 5-FU treated mice with the lymphangiogenesis inhibitor SAR131675, we no longer observed expansion of bone lymphatic vessels, as demonstrated via imaging of LYVE1-expressing lymphatic vessels and ELISA quantification of PROX1 concentration (Figure 3D). Together, these findings demonstrate that genotoxic stress leads to expansion of lymphatic vessels in the bone.

### Lymphatic vessels in bones support hematopoietic regeneration

Since genotoxic stress impacts vasculature, we examined the relationship between these two processes. We first asked whether the ability of mice to undergo lymphangiogenesis impacted hematopoietic regeneration. We compared hematopoietic reconstitution in irradiated mice with or without treatment with SAR131675. Analysis of cellular frequencies after radiation showed a decline in the number of bone marrow cells, lineage (Lin)^−^ Sca-1^+^ c-Kit^+^ (LSK) cells, and HSCs in SAR131675-treated mice (Figure 3E). We confirmed the decreases in HSC frequency by competitive secondary transplantation of bone marrow cells. Bone marrow cells from radiation and SAR131675-treated mice showed a significantly reduced reconstituting activity compared to bone marrow cells from radiation-only treated mice (Figure 3F). To interrogate whether the observed loss of cellularity and reconstitution activity in the lymphangiogenesis inhibitor-treated mice is due to defective recruitment of transplanted bone marrow cells to bone, we analyzed the recruitment after transplantation with and without inhibitor. Toward this, we performed qPCR analysis of *Tomato* expression after 24 and 36 h of transplantation of bone marrow cells from mTmG mice. This analysis showed that lymphangiogenesis inhibitor does not impact the recruitment of transplanted bone marrow cells to bone after genotoxic stress (Figure S5H). Above results suggested that inhibition of lymphangiogenesis reduces HSC regeneration capability.

To further examine whether the presence of lymphatic vessels affected HSC regeneration, we employed an alternative strategy to target LECs directly. The *Prox1-Cre ER*^*T2*^ mouse line^37^ crossed to the reporter *Rosa26-td-Tomato*, allowing visualization of PROX1-expressing lymphatic vessels. By intercrossing *Prox1 Cre ER*^*T2*^ mice with mice expressing a tamoxifen-inducible diphtheria toxin antigen (iDTA), we could specifically deplete LECs. Tamoxifen injections were performed in both Cre^−^ and Cre^+^ mice. As expected, Cre^+^ iDTA mice demonstrated suppressed expansion of the lymphatic endothelium in irradiated bones, leading to reduced PROX1 expression (Figure 3G). Consistent with the above results, Cre^+^ iDTA mice also exhibited lower bone marrow cellularity, and reduced LSK and HSC frequency, compared to Cre^−^ iDTA mice (Figure 3H). In agreement with these results, competitive transplantation of bone marrow cells from these mice confirmed significantly reduced reconstituting activity of bone marrow cells derived from Cre^+^ iDTA donors compared to Cre^−^ iDTA mice (Figure 3I). Importantly, although depletion of LECs during radiation impacted HSC frequency in Cre^+^ iDTA mice, the depletion of LECs in unirradiated Cre^+^ iDTA mice did not (Figure 3J). Similarly, treatment with SAR131675 in adult, wild-type, and unirradiated mice did not alter HSC frequency (Figure 3J). Thus, inhibition of lymphangiogenesis or loss of LECs reduces HSC regeneration after myeloablation, but HSCs are unaffected by depletion of LECs during homeostasis.

### IL6 is required for radiation-induced lymphangiogenesis in the bone

**Figure 4:**
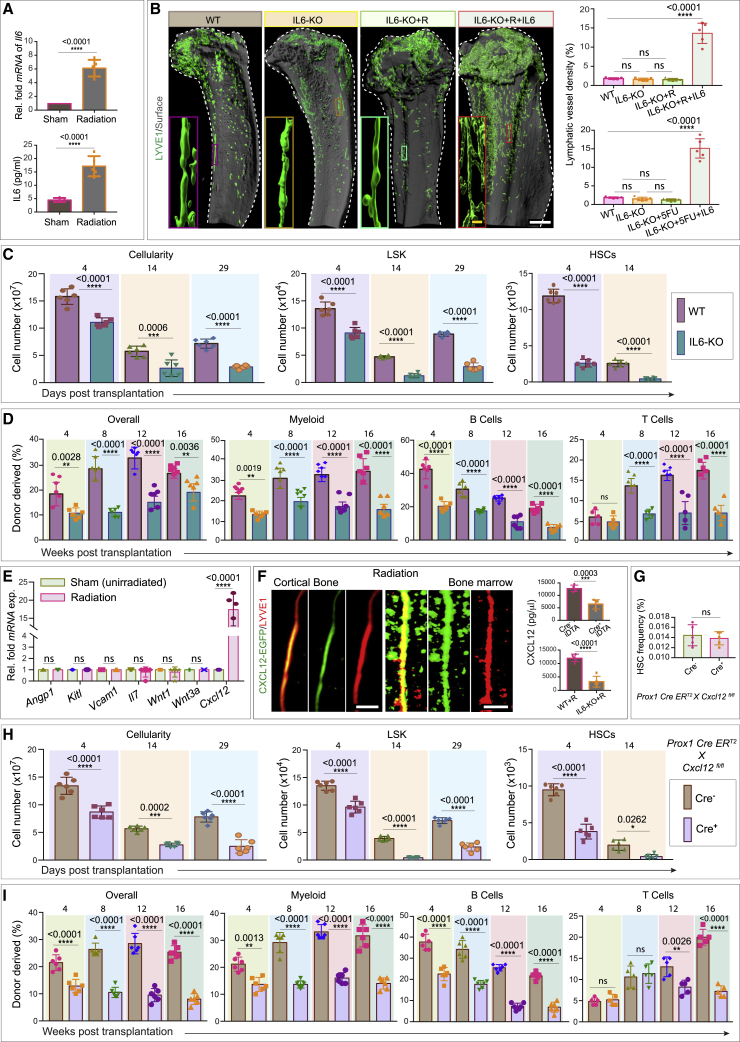
Injury-induced IL6 drives lymphangiogenesis in bone, and lymphangiocrine CXCL12 is required for hematopoietic regeneration (A) Relative fold *mRNA* expression of *Il6* and IL6 concentration in bones from radiation-treated and unirradiated (sham) mice (n = 5). (B) Tibiae showing LYVE1 immunostaining in IL6-knockout (KO) or IL6-KO + radiation (R) or IL6-KO + R + IL6-treated mice relative to wild-type (WT) mice. Quantification of lymphatic vessel density in these mice (n = 5). Quantification of lymphatic vessel density in tibiae from IL6-KO or IL6-KO + 5-fluorouracil (5-FU) or IL6-KO + 5-FU + IL6-treated mice relative to WT mice (n = 5). (C) Bone marrow cellularity, number of LSK cells, and HSCs in bones from IL6-KO or WT mice were analyzed at 4, 14, and 29 days post-radiation and BM transplantation (n = 6). (D) 1 × 10^6^ donor BM cells from WT or IL6-KO primary donor mice (as detailed in C) were transplanted into secondary WT recipient mice. Overall, myeloid, B cells, and T cells reconstitution were assessed at 4, 8, 12, and 16 week post-radiation and BM transplantation (n = 6 for overall, myeloid, and B cells; n = 5 for T cells). (E) Relative fold *mRNA* expression of *Angp1*, *Kitl*, *Vcam1*, *II7*, *Wnt1*, *Wnt3a*, and *Cxcl12* in purified bone LECs isolated from mice treated with radiation compared to unirradiated (sham) mice (n = 5). (F) Representative 3D images of tibiae from *CXCL12-EGFP* mice 10 days post-radiation. CXCL12 concentration was quantified in *Prox1 Cre ER*^*T2*^*X iDTA* bones from Cre^−^ iDTA and Cre^+^ iDTA mice after radiation (n = 5) and irradiated (R) WT and IL6-KO mice (n = 5). (G) Frequency of HSCs in unirradiated bones from Cre^−^ and Cre^+^, *Prox1 Cre ER*^*T2*^*X Cxcl12*^*fl/fl*^ mice (n = 5). Analysis was performed 29 days post-tamoxifen injections in Cre^−^ and Cre^+^ mice. (H) Bone marrow cellularity, number of LSK cells, and HSCs in bones from *Prox1 Cre ER*^*T2*^*X Cxcl12*^*fl/fl*^ mice. Cre^+^ and Cre^−^ mice were analyzed at 4, 14, and 29 days post-radiation and BM transplantation (n = 6). (I) 1 × 10^6^ donor BM cells from *Prox1 Cre ER*^*T2*^*X Cxcl12*^*fl/fl*^, Cre^+^ or Cre^−^ primary donor mice (as detailed in H) were transplanted into secondary WT recipient mice. Overall, myeloid, B cells, and T cells reconstitution were assessed at 4, 8, 12, and 16 week post-radiation and BM transplantation (n = 6 for overall, myeloid, and B cells; n = 5 for T cells). Two-tailed unpaired t tests (A, E, F, and G); one-way ANOVA with Tukey’s multiple comparisons tests (B); two-way ANOVA with Sidak multiple comparisons tests (C, D, H, and I). ^ns^p > 0.05, ^∗^p < 0.05, ^∗∗^p < 0.01, ^∗∗∗^p < 0.001, and ^∗∗∗∗^p < 0.0001. Scale bars: white, 500 μm; yellow, 80 μm. n represents biological replicates. See also Figures S5 and S6.

VEGF-C is a known critical driver of lymphangiogenesis.^23^^,^^54^^,^^55^ SAR131675 inhibits VEGF-C/VEGFR3 signaling, and the above data therefore indicate that VEGF-C/VEGFR3 signaling is involved in lymphangiogenesis in the bone during injury-induced regeneration. To gain further insight into the molecular underpinning of lymphangiogenesis in the bone, we also examined interleukin 6 (IL6), another known driver of lymphangiogenesis.^56^^,^^57^^,^^58^ Analysis of *Il6* mRNA expression levels showed a significant increase in *Il6* expression after irradiation (Figure 4A). Furthermore, ELISA analysis of bone supernatants revealed a corresponding increase in IL6 protein expression upon irradiation (Figure 4A), suggesting a role of this cytokine in bone lymphangiogenesis.

To determine whether IL6 was required for lymphangiogenesis, we examined IL6-knockout (KO) mice. The IL6-KO mice lacked expansion of lymphatic vessels after radiation or 5-FU treatment (Figure 4B). Interfering with the IL6 signaling by blocking the IL6 receptor (IL6R) in the wild-type mice inhibited lymphatic vessel expansion induced by genotoxic stress (Figure S5I). Administration of IL6 in IL6-KO mice after irradiation or 5-FU treatment mice rescued this defect, restoring lymphangiogenesis and lymphatic vessel expansion and confirming that the defects were due to the loss of IL6 (Figure 4B). As expected, given the effect on lymphangiogenesis, IL6-KO mice after myeloablation also exhibited significantly lower bone marrow cellularity and reduced LSK and HSC numbers compared to WT mice (Figure 4C). Competitive secondary transplantation of bone marrow cells from IL6-KO mice confirmed the significantly reduced reconstituting activity compared to those from wild-type mice (Figure 4D). Together, these data establish that bone lymphangiogenesis requires IL6.

Finally, we examined whether other cytokines also contribute to bone lymphangiogenesis. We found that *Il18*, *Il27*, and *Il7* are also significantly upregulated after radiation injury (Figure S5J). However, blocking of IL18, IL27, and IL7 during radiation did not interfere with lymphatic expansion as indicated by ELISA quantification of PROX1 expression (Figure S5K). Taken together, our data show that IL6-driven lymphangiogenesis plays a critical role in hematopoietic regeneration.

### LEC-derived CXCL12 supports hematopoietic regeneration

The above data demonstrated that lymphatic vessels play a critical role during hematopoietic regeneration. We next wished to determine how lymphatic vessels support hematopoietic regeneration after myeloablation. Recent evidence has shown that LECs can regulate other cell types through secreted factors known as lymphangiocrine signals.^59^^,^^60^^,^^61^^,^^62^ We therefore began by analyzing the expression of secreted factors in purified LECs from irradiated and sham mice bones. We focused on factors known to be critical for HSCs’ maintenance and survival. We observed significant upregulation of *Cxcl12* mRNA (but not other factors) in LECs after irradiation (Figure 4E). Indeed, analysis of CXCL12-EGFP mice showed CXCL12-positive lymphatic vessels in bones from irradiated mice (Figure 4F). Importantly, inhibition of lymphangiogenesis in *Prox1 Cre*^*+*^ *iDTA* mice, or in IL6-KO mice, prevented this upregulation of CXCL12 in the bone marrow supernatant after radiation injury (Figure 4F), demonstrating that upregulation of CXCL12 depends on the presence of LECs and on lymphangiogenesis and is not produced by other cells in the bone. CXCL12 is therefore a candidate lymphangiocrine signal.

**Figure S6:**
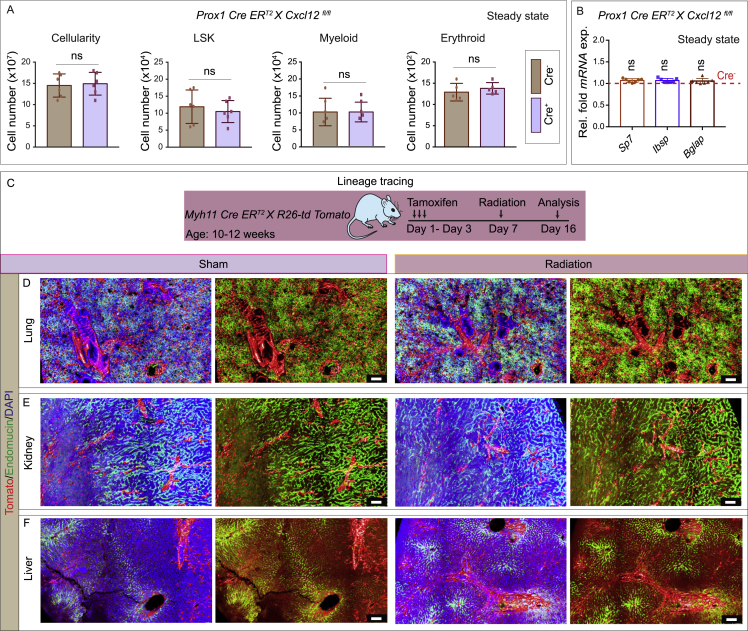
Quantification of hematopoietic cells, expression of osteogenic markers in steady-state *Prox1 Cre ER*^*T2*^*X Cxcl12*^*fl/fl*^ mice and lineage tracing of Myh11^+^ pericytes across different organs, related to Figures 4 and 5 (A) Bone marrow cellularity (left panel), number of LSK cells (center-left panel), myeloid (center-right panel) and erythroid (right panel) in Cre^+^ and Cre^−^, *Prox1 Cre ER*^*T2*^*X Cxcl12*^*fl/fl*^ mice bones were analyzed at steady state (*n* = 6 biological replicates for cellularity and LSK; *n* = 5 biological replicates for myeloid and erythroid). Analysis was performed 29 days post-tamoxifen injections in Cre^+^ mice and Cre^−^ littermate controls. These analyses were performed at steady state/homeostasis. Statistical significance between each group was determined using two-tailed unpaired t-tests (^ns^p>0.05). (B) Relative fold *mRNA* expression of *Sp7*, *Ibsp* and *Bglap* in tibial bones from Cre^+^*Prox1 Cre ER*^*T2*^*X Cxcl12*^*fl/fl*^ compared with their Cre^−^ littermate controls. These analyses were performed at steady state/homeostasis (*n* = 7 biological replicates). Analysis was performed 29 days post-tamoxifen injections in Cre^−^ and Cre^+^ mice. p values derived from two-tailed unpaired t-tests (^ns^p>0.05). (C) Schematic depicting the experimental design, whereby tamoxifen-inducible *Myh11 Cre ER*^*T2*^*X R26-td Tomato* mice, were subjected to radiation 7 days post-tamoxifen administration, and organs were subsequently collected and stained 10 days post-radiation treatment. (D-F) Confocal images of (D) lung, (E) kidney, and (F) liver tissues from *Myh11 Cre ER*^*T2*^*X R26-td Tomato*, organs from radiation treated (right panels) and unirradiated sham (left panels) mice; Tomato (red), Endomucin (green) and DAPI (blue). Scale bars: 50 μm.

To determine the functional significance of CXCL12 expression, we examined the bone marrow of LEC-specific CXCL12 loss-of-function mice. Unirradiated *Prox1 Cre ER*^*T2*^ *X Cxcl12* ^*fl/fl*^ mice had normal bone marrow cellularity and HSC, LSK, myeloid, and erythroid cell numbers (Figures 4G and S6A). They also exhibited normal expression of bone and bone progenitor markers (Figure S6B), demonstrating that loss of CXCL12 did not impact the bone marrow under homeostatic conditions. However, after myeloablation and transplantation with bone marrow cells from wild-type mice, LEC-specific CXCL12 loss-of-function mice had significantly lower bone marrow cellularity and reduced numbers of LSK cells and HSCs compared to Cre^−^ mice (Figure 4H). Further, competitive secondary transplantation of bone marrow cells from LEC-specific CXCL12 loss-of-function mice showed lower hematopoietic reconstitution ability (Figure 4I). Thus, CXCL12 acts as a lymphangiocrine signal to promote hematopoietic regeneration.

### Lymphatic vessels promote bone regeneration by promoting the expansion of Myh11-positive pericytes

**Figure 5:**
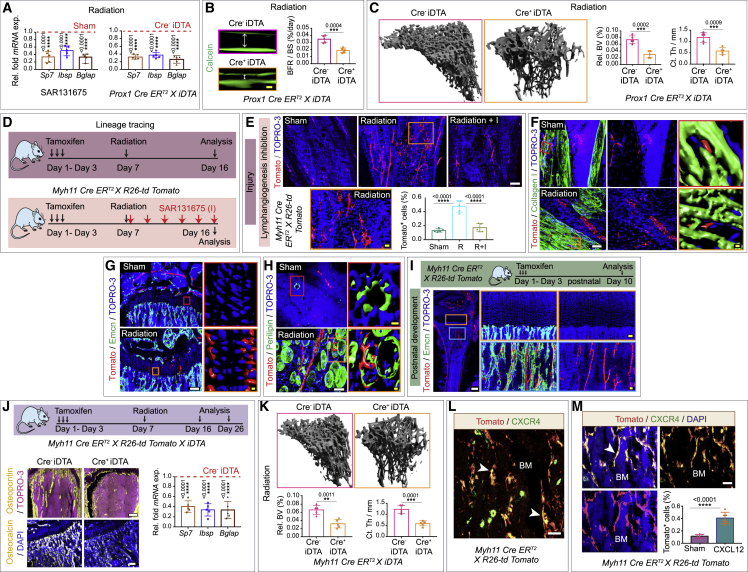
Lymphatic vessels drive bone regeneration via Myh11-positive pericytes (A) Relative fold *mRNA* expression of *Sp7*, *Ibsp*, and *Bglap* in wild-type mice treated with radiation- and SAR131675-treated compared to sham mice treated with radiation alone (n = 5). Relative fold *mRNA* expressions in radiation-treated *Prox1 Cre ER*^*T2*^*X iDTA*; Cre^+^ iDTA mice relative to their Cre^−^ iDTA littermate control mice (n = 5). (B) Bone formation rate per bone surface (BFR/BS) in radiation-treated Cre^−^ iDTA and Cre^+^ iDTA, *Prox1 Cre ER*^*T2*^*X iDTA* mice; calcein (green). Data represent mean ± SEM (n = 5). (C) Representative μ-CT images of tibiae from radiation-treated Cre^−^ iDTA and Cre^+^ iDTA, *Prox1 Cre ER*^*T2*^*X iDTA* mice. Quantitative analysis of relative bone volume (Rel. BV.) and cortical thickness (Ct. Th.) of tibiae (n = 5). (D) Schematic depicting the experimental design, whereby various tamoxifen-inducible Cre mouse strains were subjected to radiation 1 week post-tamoxifen, and tibiae were subsequently collected at 10 days post-radiation. In some cases, mice were additionally treated with a VEGFR3 inhibitor (SAR131675: I) immediately after radiation and for every successive 48 h up until 10 days. (E) 3D images of tibiae showing Tomato and TOPRO-3 from *Myh11 Cre ER*^*T2*^*X R26-td Tomato* mice treated with radiation (R), radiation along with SAR131675 (I) inhibitor (R + I), and unirradiated PBS-treated (sham) mice with a high-magnification inset. Quantification of Tomato-positive cells in the bones from *Myh11 Cre ER*^*T2*^*X R26-td Tomato* mice (n = 5). (F) 3D images of tibiae from unirradiated sham and radiation-treated mice with Tomato, collagen I, and TOPRO-3. (G) 3D images of tibiae with Tomato, endomucin (Emcn), and TOPRO-3 from unirradiated sham and radiation-treated mice with high-magnification insets from growth plate regions. (H) 3D images of tibiae showing Tomato, perilipin, and TOPRO-3 from unirradiated sham and radiation-treated mice with high-magnification insets. (I) Schematic depicting the experimental design, whereby both Cre^+^ and Cre^−^, *Myh11 Cre ER*^*T2*^*X R26-td Tomato* mice were subjected to tamoxifen treatment at postnatal day 1, and tibiae were subsequently collected after 10 days. Representative 3D images of a Cre^+^ tibia showing Tomato expresssion pattern. (J) Schematic depicting the experimental design, whereby various tamoxifen-inducible *Myh11 Cre ER*^*T2*^*X R26-td Tomato X iDTA* mice were subjected to radiation 1 week post-tamoxifen treatment, and tibiae were subsequently collected at 10 days or 20 days post-radiation. 3D images from radiation-treated Cre^−^ iDTA or Cre^+^ iDTA mice; labeled with osteopontin and osteocalcin. Relative fold *mRNA* expression of *Sp7*, *Ibsp*, and *Bglap* in *Myh11 Cre ER*^*T2*^*X R26-td Tomato X iDTA* mice treated with radiation; Cre^+^ iDTA mice as compared to their Cre^−^ iDTA littermate control mice (n = 5). (K) 3D μ-CT images of tibiae from radiation-treated Cre^−^ iDTA and Cre^+^ iDTA, *Myh11 Cre ER*^*T2*^*X iDTA* mice. Analysis of relative bone volume (Rel. BV.) and cortical thickness (Ct. Th.) of tibiae (n = 5). (L) 3D image of a tibiae from *Myh11 Cre ER*^*T2*^*X R26-td Tomato* mice showing Tomato and CXCR4 in bone marrow (BM). Arrowheads, Tomato and CXCR4 double-positive cells. (M) 3D images of a tibia from *Myh11 Cre ER*^*T2*^*X R26-td Tomato* mice showing Tomato, CXCR4, and DAPI. Quantification of Tomato-positive cells from the CXCL12-treated and PBS-injected (sham) mice (n = 6). Arrowhead showing Tomato and CXCR4 double-positive cells. Two-tailed unpaired t tests (A–C, J, K, and M); one-way ANOVA tests with Tukey’s multiple comparisons tests (E). ^∗∗^p < 0.01, ^∗∗∗^p < 0.001, and ^∗∗∗∗^p < 0.0001. Scale bars: white, 50 μm; yellow, 10 μm. n represents biological replicates. See also Figures S6, S7, and S8.

We next investigated whether lymphatic vessels also play a role in bone regeneration and bone formation after irradiation by comparing the expression of bone progenitor and osteoblast cell markers in LEC-depleted and sham mice. We found reduced expression of osteoprogenitor and osteoblast markers in long bones of irradiated mice treated with SAR131675 or in *Prox1 Cre*^*+*^ *iDTA* mice, compared to sham mice or *Prox1 Cre*^*−*^ *iDTA* mice (Figure 5A). Calcein double labeling showed reduced bone formation rates after genotoxic stress in LEC-depleted mice, e.g., *Prox1 Cre*^*+*^ *iDTA* mice compared to Cre^−^ iDTA mice (Figure 5B). Furthermore, microcomputed tomography (μ-CT) showed that LEC depletion, e.g., in *Prox1 Cre*^*+*^ *iDTA* mice, led to significantly decreased bone mass in regenerating bones after irradiation injury (Figure 5C). These data suggested that lymphangiogenesis contributes to bone regeneration after irradiation.

**Figure S7:**
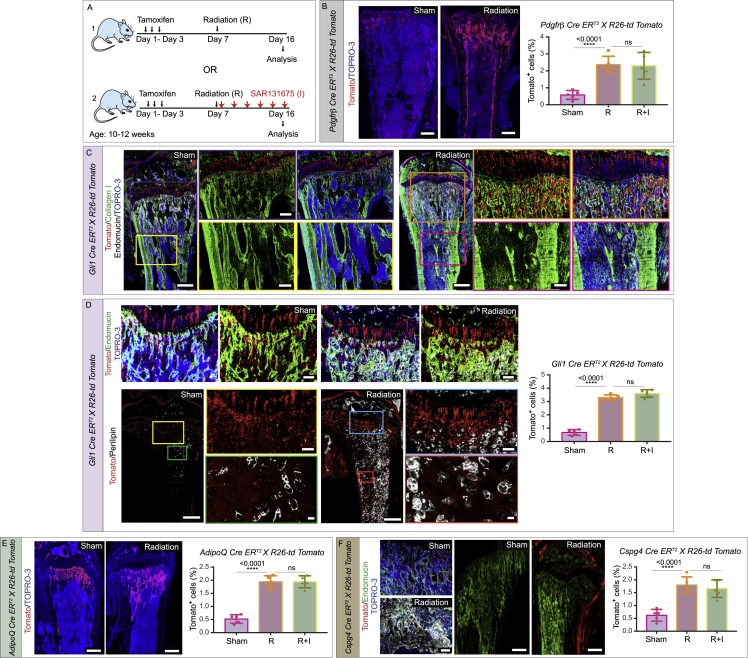
Lineage tracing of various mesenchymal cells in bones during homeostasis and genotoxic stress, related to Figure 5 (A) Schematic depicting the experimental design, whereby various tamoxifen-inducible Cre mouse strains were subjected to radiation 1-week post-tamoxifen treatment. At the end of the radiation (R), the mice received an injection of the VEGFR3 inhibitor (SAR131675: I), and tibial bones were subsequently collected and stained for imaging. All experiments were performed with *n* = 6 biological replicates. (B) Immunostaining of the murine tibial bones of *Pdgfrβ Cre ER*^*T2*^*X R26-td Tomato*, healthy mice (sham) or mice subjected to radiation; Tomato (red) and TOPRO-3 (blue). Quantification of Tomato^+^ cells in tibial bones from R, R + I, and unirradiated PBS treated sham *Pdgfrβ Cre ER*^*T2*^*X R26-td Tomato* mice. Scale bars: 300 μm. (C) Immunostaining of the murine tibial bones of *Gli1 Cre ER*^*T2*^*X R26-td Tomato*, unirradiated mice (sham) or mice subjected to radiation with high magnification insets; Tomato (red), Collagen I (green), Endomucin (white), and TOPRO-3 (blue). Scale bars: 300 μm and insets 50 μm. (D) Immunostaining of the murine tibial bones of *Gli1 Cre ER*^*T2*^*X R26-td Tomato*, unirradiated mice (sham) or mice subjected to radiation; Tomato (red), Endomucin (green), TOPRO-3 (blue) and Perilipin (white). Quantification of Tomato^+^ cells from *Gli1 Cre ER*^*T2*^*X R26-td Tomato* mice. Scale bars: 50 μm (upper panels); 500 μm (lower panel) and insets 50 μm (yellow and blue) or 10 μm (green and red). (E) Immunostaining of the murine tibial bones of *AdipoQ Cre ER*^*T2*^*X R26-td Tomato* unirradiated mice (sham) or mice subjected to radiation; Tomato (red) and TOPRO-3 (blue). Quantification of Tomato^+^ cells from *AdipoQ Cre ER*^*T2*^*X R26-td Tomato* mice. Scale bars: 500 μm. (F) Immunostaining of the murine tibial bones of *Cspg4 Cre ER*^*T2*^*X R26-td Tomato* unirradiated mice (sham, top-left or center panel) or mice subjected to radiation (bottom-left and right panel); Tomato (red), Endomucin (green) and TOPRO-3 (blue). Bar graph shows the quantification of Tomato^+^ cells from *Cspg4 Cre ER*^*T2*^*X R26-td Tomato* mice. Scale bars: 50 μm (left) and 500 μm (right). (B-F) p values derived from one-way ANOVA tests with Tukey’s multiple comparisons tests (*n*= 5). (^ns^p>0.05 and ^∗∗∗∗^p < 0.0001).

We therefore sought to determine which specific bone cell progenitor population was impacted by the inhibition of lymphangiogenesis. Through lineage tracing approach with multiple mesenchymal and perivascular cell markers, we found that irradiation induced a significant expansion of myosin heavy-chain 11 (Myh11)-positive cells in bones but not in other organs (Figures 5D, 5E, and S6C–S6F). Myh11 cells also express alpha smooth muscle actin (α-SMA)^63^^,^^64^; these Myh11-positive cell populations represent mature pericytes. After irradiation, these cells gave rise to chondrocyte, adipocyte, and osteoblast lineages (Figures 5D and 5F–5H). However, expansion and differentiation of this Myh11-positive cell population was not observed upon inhibition of lymphangiogenesis (treatment with SAR131675) (Figures 5D and 5E). In addition, lineage tracing of Myh11 cells during normal bone development and homeostasis indicated that Myh11-positive cells remained near large arteries and did not differentiate into other lineages as during irradiation (Figure 5I). Unlike Myh11-positive cells, other mesenchymal cell types such as Pdgfrβ^+^ cells, Gli1^+^ cells, AdipoQ^+^ cells, and Cspg4^+^ cells, which are also known to contribute to bone formation or regeneration, did not show any changes in abundance upon suppression of lymphangiogenesis (Figures S7A–S7F). These results suggested that irradiation and lymphangiogenesis specifically impact Myh11-positive cells.

To test the functional relevance of the Myh11-positive cell population during bone regeneration, we crossed *Myh11 Cre ER*^*T2*^ mice with the iDTA mouse line described above to specifically deplete Myh11-positive cells. After irradiation, we observed reduced expression of osteoprogenitor and osteoblast markers in long bones of Cre^+^ iDTA mice (lacking Myh11-positive cells) compared to Cre^−^ iDTA mice (Figure 5J), suggesting that Myh11-positive cells promote bone formation after injury. Consistent with this idea, immunostaining of osteopontin, which accumulates around trabecular and cortical bones, showed fewer bony elements in irradiated, *Myh11 Cre*^*+*^ *iDTA* mice compared to Cre^−^ iDTA mice (Figure 5J). Immunostaining with osteocalcin, a marker for osteoblasts, showed reduced expression in bones from *Myh11 Cre*^*+*^ *iDTA* mice (Figure 5J), consistent with a reduction in bone elements (Figure 5J). Furthermore, μ-CT examination showed that irradiated mice with depleted Myh11-positive cells had significantly decreased bone mass (Figure 5K). These results are all consistent with the idea that Myh11-positive cells are required for bone regeneration.

**Figure 6:**
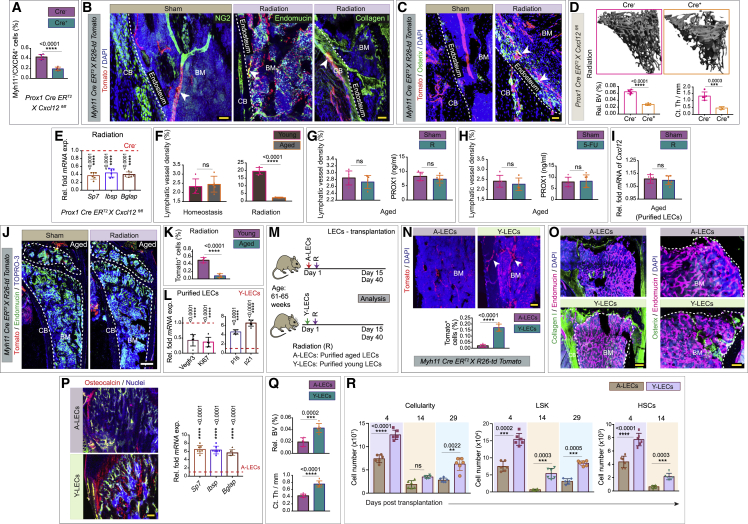
Age-dependent changes in LECs in homeostasis and during genotoxic stress (A) Fluorescence-activated cell sorting (FACS) quantification of Myh11-positive and CXCR4^+^ cells in Cre^−^ and Cre^+^, *Prox1 Cre ER*^*T2*^*X Cxcl12*^*fl/fl*^ mice (n = 6). (B) 3D images of tibiae from unirradiated (sham) and radiation-treated *Myh11 Cre ER*^*T2*^*X R26-td Tomato* mice showing Tomato, NG2, endomucin, and collagen I. Dashed lines indicate the endosteal regions of the bone. Arrowheads, Tomato-positive cells. (C) Confocal images of tibiae from unirradiated (sham) and radiation-treated *Myh11 Cre ER*^*T2*^*X R26-td Tomato* mice showing osterix, Tomato, and DAPI. Dashed lines denote the endosteal regions of the bone. Arrowheads, Tomato-positive cells. (D) μ-CT images of tibiae from radiation treated Cre^−^ and Cre^+^, *Prox1 Cre ER*^*T2*^*X Cxcl12*^*fl/fl*^ mice. Quantitative analysis of relative bone volume (Rel. BV.) and cortical thickness (Ct. Th.) of the bones (n = 5). (E) Relative fold *mRNA* expression of *Sp7*, *Ibsp*, and *Bglap* in radiation-treated Cre^+^, *Prox1 Cre ER*^*T2*^*X Cxcl12*^*fl/fl*^ mice compared to their Cre^−^ littermate controls (n = 6). (F) Quantification of lymphatic vessel density in tibiae from young and aged mice during homeostasis and after radiation (n = 5). (G) Quantification of lymphatic vessel density in tibiae from aged mice treated with radiation compared to unirradiated (sham) mice (n = 5). ELISA quantification of PROX1 concentration in tibiae from aged mice treated with radiation compared to unirradiated (sham) mice (n = 6). (H) Quantification of lymphatic vessel density in tibiae from aged mice treated with 5-FU compared to sham mice (n = 5). ELISA quantification of PROX1 concentration in tibiae from aged mice treated with 5-FU compared to PBS-injected sham mice (n = 6). (I) Relative fold *mRNA* expression of *Cxcl12* in purified lymphatic endothelial cells (LECs) isolated from bones in aged mice treated with radiation compared to unirradiated (sham) mice (n = 7). (J) 3D images of tibiae from *Myh11 Cre ER*^*T2*^*X R26-td Tomato* mice treated with radiation compared to unirradiated (sham) mice; showing Tomato and endomucin. (K) Quantification of Tomato-positive cells in tibiae from young and aged *Myh11 Cre ER*^*T2*^*X R26-td Tomato* mice after radiation (n = 5). (L) Relative fold *mRNA* expression of *Vegfr3*, *Ki67*, *p16*, and *p21* in purified LECs isolated from young (Y-LECs) and aged (A-LECs) murine tibiae (n = 6). (M) Schematic depicting the experimental design, whereby aged mice were administered with purified A-LECs or Y-LECs followed by the radiation. Bones were analyzed post-radiation at day 15 to day 40. (N) 3D images of tibiae from *Myh11 Cre ER*^*T2*^*X R26-td Tomato* aged mice transplanted with A-LECs or Y-LECs and subsequently subjected to radiation (as detailed in M) showing Tomato and DAPI. Arrowheads show the expansion of Tomato-positive cells in aged mice transplanted with Y-LECs. Quantification of Tomato-positive cells in aged bones isolated from mice transplanted with A-LECs and Y-LECs (n = 5). (O) 3D images of aged mice tibiae after A-LECs or Y-LECs transplantation and radiation showing immunostaining for collagen I, endomucin, and osterix. (P) 3D images of tibiae from aged mice with the transplantation of A-LECs or Y-LECs and radiation showing immunostaining for osteocalcin. Nuclei were stained with DAPI. Relative fold *mRNA* expression of *Sp7*, *Ibsp*, and *Bglap* in tibia from irradiated mice transplanted with Y-LECs as compared to the mice transplanted A-LECs (n = 7). (Q) Analysis of relative bone volume (Rel. BV.) and cortical thickness (Ct. Th.) in aged mice subjected to radiation and transplanted with A-LECs or Y-LECs (n = 6). (R) Bone marrow cellularity, number of LSK cells, and HSCs in bones from aged mice transplanted with A-LECs or Y-LECs after radiation. Analyses were performed at 4, 14, and 29 days post-transplantation (n = 6). Two-tailed unpaired t tests (A, D–I, K, L, N, P, and Q); two-way ANOVA with Sidak multiple comparisons tests (R). ^ns^p > 0.05, ^∗∗^p < 0.01, ^∗∗∗^p < 0.001, and ^∗∗∗∗^p < 0.0001. Scale bars: white, 300 μm; yellow, 30 μm. n represents biological replicates. Bone marrow, BM; cortical bone, CB. See also Figure S8.

As shown above, after irradiation, LECs express CXCL12, and this chemokine acts as a lymphangiocrine factor to promote HSC reconstitution in the bone. CXCR4 is the CXCL12 receptor. We found that Myh11-positive cells express CXCR4, as demonstrated by CXCR4 immunostaining on bone sections in *Myh11 Cre ER*^*T2*^ *X R26-td Tomato* mice (Figure 5L), raising the possibility that Myh11-positive cells could respond to CXCL12 signaling from LECs. Indeed, administration of CXCL12 in mice led to the expansion of the Myh11-positive cell population in bones even in the absence of genotoxic stress (Figure 5M). Interestingly, analysis of *Prox1 Cre ER*^*T2*^ *X Cxcl12* ^*fl/fl*^ mouse bones demonstrated a significant decrease in the Myh11-positive and CXCR4^+^ cells as quantified by flow cytometry (Figure 6A), indicating that CXCL12 derived from lymphatic vessels is required for the expansion of Myh11^+^ CXCR4^+^ cells.

**Figure S8:**
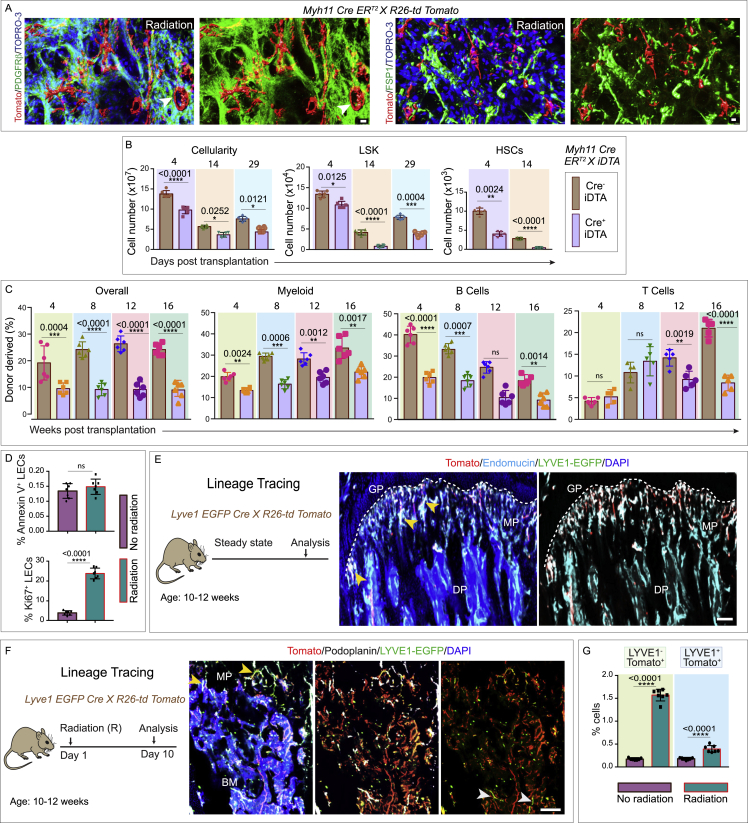
Lineage tracing and analysis of Myh11^+^ pericytes and LECs in homeostasis versus genotoxic stress in bones, related to Figures 5 and 6 (A) Representative 3D images of tibial bones from *Myh11 Cre ER*^*T2*^*X R26-td Tomato* mice showing Tomato (red), PDGFRβ (green, left panels), and FSP1 (green, right panels), Nuclei were stained with TOPRO-3 (blue). Arrowheads show the adipocyte forming Tomato^+^ cells. Scale bars: 15 μm (left) and 10 μm (right). (B) Bone marrow (BM) cellularity (left panel), number of LSK cells (center panel) and HSCs (right panel) in tibial bones from Cre^−^ iDTA and Cre^+^ iDTA, *Myh11 Cre ER*^*T2*^*X iDTA* mice were analyzed at 4-, 14- and 29- days post-radiation treatment and BM transplantation (*n* = 6 biological replicates). Statistical significance between each group was determined using two-way ANOVA with Sidak multiple comparisons tests (^∗^p < 0.05, ^∗∗^p < 0.01, ^∗∗∗^p < 0.001 and ^∗∗∗∗^p < 0.0001). (C) 1 x 10^6^ donor BM cells from Cre^−^ iDTA and Cre^+^ iDTA, *Myh11 Cre ER*^*T2*^*X iDTA* treated primary donor mice (as detailed in B) were transplanted into secondary wild type recipient mice 4 weeks after radiation treatment. Overall (left panel), myeloid (center-left panel), B cells (center-right panel) and T cells (right panel) reconstitution were assessed at 4-, 8-, 12- and 16-week post-radiation treatment and BM transplantation (*n* = 6 biological replicates for overall, myeloid and B cells; *n* = 5 biological replicates for T cells). Statistical significance between each group was determined using two-way ANOVA with Sidak multiple comparisons tests (^ns^p>0.05, ^∗∗^p < 0.01, ^∗∗∗^p < 0.001 and ^∗∗∗∗^p < 0.0001). (D) FACS quantification of Annexin V^+^ LECs (top panel) in unirradiated versus irradiated mice. (*n* = 7 biological replicates). p value derived from a two-tailed unpaired t-test (^ns^p>0.05). Further, FACS quantification of Ki67^+^ LECs (bottom panel) in unirradiated versus irradiated mice (*n* = 7 biological replicates). p value derived from a two-tailed unpaired t-test (^∗∗∗∗^p < 0.0001). (E) Schematic depicting the experimental design, whereby *Lyve1 EGFP Cre X R26-td Tomato* mice were generated. Bones were collected for analysis at the age of 10-12 weeks at steady state. Representative 3D images of the tibial bone showing Tomato (red), LYVE1-EGFP (green), Endomucin immunostaining (cyan), and nuclear staining with DAPI (blue). Arrowheads indicate Tomato positive type H vessels in the metaphysis (MP) region of the bone. Note the tomato expression in type H vessels in metaphysis but not in the sinusoidal vessels in diaphysis. Growth plate: GP; diaphysis: DP. Scale bar: 50 μm. (F) Schematic depicting the experimental design, whereby *Lyve1* EGFP *Cre X R26-td Tomato* mice were subjected to radiation prior to bone collection. 3D images showing Tomato (red), LYVE1-EGFP (green), Podoplanin (white) and DAPI (blue). Yellow arrowheads indicate the Tomato and LYVE1-EGFP double-positive cells. White arrowheads show LYVE1-EGFP positive but Tomato negative cells. (Metaphysis: MP; bone marrow: BM). Scale bar: 150 μm. (G) FACS quantification of LYVE1-EGFP^-^ Tomato^+^ cells and LYVE1-EGFP^+^ Tomato^+^ in tibial bones from *Lyve1 EGFP Cre X R26-td Tomato* unirradiated mice versus mice subjected to radiation treatment (*n* = 7 biological replicates). p value derived from a two-tailed unpaired t-test (^∗∗∗∗^p < 0.0001).

Further, tracking and lineage tracing of Myh11-positive cells demonstrated that they expand in both the bone marrow, where they differentiate into adipocytes, and the endosteal region of bones, where they differentiate into perivascular osteoblasts (Figures 5H, 6B, 6C, and S8A). Vasculature and osteoblasts in endosteal regions are known to be niche for HSC and critical sites for hematopoietic regeneration.^6^^,^^7^^,^^11^^,^^65^^,^^66^ In line with this, *Myh11 Cre ER*^*T2*^ *X iDTA*, Cre^+^ iDTA mice with depletion of Myh11-positive cells exhibited lower bone marrow cellularity and reduced LSK and HSC frequencies post-radiation (Figure S8B). Competitive transplantation of bone marrow cells from these mice confirmed significantly reduced reconstituting activity by bone marrow cells derived from Cre^+^ iDTA donors compared to Cre^−^ iDTA donors (Figure S8C).

Next, we wondered whether CXCL12 may also mediate the effects of lymphangiogenesis on bone formation during genotoxic stress. This investigation revealed that the loss of CXCL12 expression in the lymphatic endothelium as in *Prox1 Cre ER*^*T2*^ *X Cxcl12* ^*fl/fl*^ mice led to a decrease in bone mass and reduced expression of osteogenesis markers after radiation (Figures 6D and 6E). Together, these data demonstrate that Myh11-positive cells are required for hematopoietic and bone regeneration and that the lymphangiocrine factor CXCL12 drives their expansion after genotoxic stress.

### Aging inhibits the response of LECs to genotoxic stress

Aging, which is associated with alterations in the bone marrow microenvironment, is known to impact bone and HSC regeneration. Given the role of LECs and lymphangiogenesis in bone and HSC regeneration that we showed above, we performed a series of experiments to test whether changes in lymphangiogenesis may contribute to aging-associated reduced regeneration abilities in the bone. Unlike bones from young mice, bones from aged mice did not exhibit lymphatic vessel expansion in response to genotoxic stress (Figures 6F–6H). Further, unlike the young mice, LECs purified from the bones of aged mice after radiation do not upregulate *Cxcl12* (Figures 4E and 6I). In line with this finding, there is no expansion of Myh11-positive cells after radiation in aged mice as demonstrated by imaging of tibial bones (Figure 6J). Also, we quantified Myh11-positive cells in young versus aged mice bones, which confirmed lack of expansion of these Myh11-positive cells in aged bones after irradiation (Figure 6K). Thus, the responses of LECs and Myh11-positive cells to genotoxic stress change with aging. Finally, to understand the consequence of genotoxic stress on LECs, we performed apoptosis and proliferation analysis during radiation. Interestingly, LECs were damage resistant to genotoxic stress as illustrated by lack of changes upon radiation in the frequency of apoptotic LECs; at the same time, there was a dramatic increase in the proliferation of LECs (Figure S8D). Further, lineage analysis with *Lyve1 EGFP Cre X R26-td Tomato* mice led to a surprising finding that the type H vessels in the bones have lymphatic origin during development as demonstrated by the presence of Tomato-positive, Endomucin^+^, LYVE1-EGFP^−^ column-like vessels in the adult mouse bones (Figure S8E). Type H endothelial cells represent the angiogenic subset of endothelial cells in bone and respond to radiation injury to recover the blood vasculature from radiation stress.^12^ As expected, after the radiation due to rapid proliferation of type H endothelial cells, there was a rapid increase in Tomato-positive cells, of which only a small subset was LYVE1-positive and Tomato-positive, indicating that a small fraction of LECs during genotoxic stress have type H origin (Figures S8F and S8G).

Next, we asked whether senescence could explain the loss of regenerative ability in LECs and bone cells from aged mice. We therefore analyzed the expression of senescence markers in sorted LECs from young and aged mice. qPCR analysis of senescence markers showed upregulation of *p16* and *p27* (Figure 6L) in aged mice LECs. Moreover, the LECs from aged mice demonstrated downregulation of proliferation marker *Ki67* and lymphatic endothelial marker *Vegfr3* (Figure 6L). These data suggested that cell-intrinsic changes during aging may drive the lack of response to the genotoxic stress by LECs in aged mice as observed above. If that is the case, we reasoned that transplantation of LECs from young to aged mice might restore regenerative abilities. To test this hypothesis, we injected LECs into the bones of aged mice via intra tibial route, prior to radiation (Figure 6M). Administration of LECs isolated from young but not aged mice led to substantial expansion of Myh11-positive cells (Figure 6N) in irradiated aged mice and increased expression of several bone markers, including collagen I, osterix, and osteocalcin (Figures 6O and 6P). The expression of osteoprogenitor and osteoblast markers also increased significantly in aged mice injected with young LECs after radiation injury (Figure 6P). Furthermore, μ-CT examination showed that administration of young LECs led to significantly increased bone mass (Figure 6Q). Moreover, aged mice injected with young LECs showed increased hematopoietic regeneration after genotoxic stress relative to mice injected with aged LECs (Figure 6R). Thus, aging leads to impairment in genotoxic stress-induced lymphatic expansion and lymphangiocrine signaling due to cell-intrinsic aging of LECs, and the administration of young LECs promotes bone and hematopoietic regeneration in aged bones.

## Discussion

In this work, we modified the existing methods to image intact skeletal tissue at a high resolution. Using in-depth 3D imaging, we establish the presence of lymphatic vessels in murine and human bones. We show that radiation- and chemotherapy-induced injury promote lymphangiogenesis and expansion of lymphatic vessels in the bone, and these, in turn, support the regeneration of HSCs within the bone and the bone itself. Lymphangiogenesis requires both VEGF-C and IL6 signaling, while the lymphangiocrine factor CXCL12 mediates the effect of LECs on HSCs and bone. These effects diminish with age due to changes in the ability of LECs to expand and support regeneration.

Lymphatic vessels play a vital role in facilitating the transport of essential fluids, macromolecules, and immune cells.^16^^,^^17^^,^^67^^,^^68^^,^^69^^,^^70^^,^^71^^,^^72^^,^^73^ Recent work suggests an even broader array of functions. For instance, lymphatic vessels support cardiac development, and homeostasis, and help restore heart tissue following injury via secretion of the LEC-derived extracellular protein, relin.^37^^,^^74^^,^^75^^,^^76^^,^^77^^,^^78^ In the skin, lymphatic vessel-stem cell crosstalk drives wound repair.^79^ Specifically, in the skin context, stem cells support lymphatic drainage and wound repair through secretion of the lymphangiocrine factor, angiopoietin-like protein 7 (Angptl7).^80^ LECs have also been implicated in cancer metastasis,^81^^,^^82^ where the LEC-derived ETS domain-containing protein 2 (ELK2) facilitates communication between the tumor and its surrounding microenvironment.^83^ Here, we identify a fundamental role for LECs in mediating bone and hematopoietic regeneration after genotoxic stress. Finally, our findings show that cell-intrinsic changes in the bone lymphatic endothelium during aging underlie their lack of response to genotoxic stress in aged bones, thereby impacting bone and hematopoietic regeneration in aged animals. These findings raise further questions regarding LEC function, and it would be particularly interesting to examine the potential crosstalk between lymphatic vessels and bone-resident immune cells, for instance in the context of inflammatory diseases of the bone such as rheumatoid arthritis and osteoarthritis. These findings also raise the possibility of manipulating LECs and lymphangiocrine factors in clinical settings to accelerate regeneration in the skeletal system. Furthermore, these key findings will contribute to designing therapeutic approaches to treat blood and bone diseases.

### Limitations of the study

Transplantation of young LECs boosts bone and hematopoietic regeneration in aged mice. However, we did not characterize differences between young and aged LECs. Investigating age-dependent changes in LECs will provide a more in-depth understanding of bone LECs and strategies to manipulate them. Functions of lymphatic vessels include fluid transport and immunosurveillance. In this context, the limitation of this study is that we have not investigated where the lymphatic vessels in bones drain. Characterizing the locations where these lymphatic vessels drain will add to understanding broader lymphatic physiological, associated functions and drug interventions in bone. Also, we have not studied the organ-specific differences between bone LECs versus LECs from other organs.

## STAR★Methods

### Key resources table

**Table:** 

REAGENT or RESOURCE	SOURCE	IDENTIFIER
**Antibodies**		

⍺-SMA	Sigma-Aldrich	CAT#C6198; RRID:AB_476856
Endomucin	Santa Cruz Biotechnology	CAT#sc-65495; RRID:AB_2100037
CD102	BD Pharmingen	CAT#553326 RRID: AB_394784
Collagen I	Sigma-Aldrich	CAT#AB765P RRID: AB_92259
SOX9	Abcam	CAT#ab185966 RRID: AB_2728660
SM22⍺	Abcam	CAT#ab14106
CD8α	Abcam	CAT#ab237365; N/A
CD31	BD Pharmingen	CAT#553370; RRID:AB_443021
CD31	R&D Systems	CAT#AF3628; RRID:AB_2161028
Endoglin	R&D Systems	CAT#AF1320; RRID:AB_354735
Endoglin	R&D Systems	CAT#AF1097; RRID:AB_354598
CD68	GeneTex	CAT#GTX43913; RRID:AB_11166091
Osteopontin	R&D Systems	CAT#AF808; RRID:AB_2194992
Perilipin	Cell signaling	CAT#9349; RRID:AB_10829911
IL-6 Receptor	ThermoFisher Scientific	CAT#PA5-47209; RRID:AB_2609193
Mouse IL-6 Neutralizing mAb	InvivoGen	CAT#mabg-mil6-3; N/A
Mouse IL-27 Neutralizing mAb	Bio X Cell	CAT# BE00326; N/A
mouse/human IL-7 Neutralizing mAb	Bio X Cell	CAT# BE0048; RRID:AB_1107711
GFP	ThermoFisher Scientific	CAT#A-21311; RRID:AB_221477
Podoplanin	R&D Systems	CAT#AF3244; RRID:AB_2268062
LYVE1	Abcam	CAT#ab14917; RRID:AB_301509
PROX1	Abcam	CAT#ab101851; RRID:AB_10712211
Ki67	Invitrogen	CAT#14-5698-82; RRID:AB_10854564
Ki67	Abcam	CAT#ab15580; RRID:AB_443209
CD146	Abcam	CAT#ab196448; RRID:AB_2868591
PDGF Receptor beta	Abcam	CAT#ab32570; RRID:AB_777165
CD45	BioLegend	CAT#103105; RRID:AB_312970
FABP4	R&D Systems	CAT#AF1443;RRID:AB_2102444
Heparan sulfate Proteoglycan	Millipore	CAT#MAB1948P; RRID:AB_10615958
CD144	R&D Systems	CAT#AF1002; RRID:AB_2077789
CD34	eBioscience	CAT#14-034182; RRID:AB_467210
CD11b	Abcam	CAT#ab197702; N/A
Osterix	Santa Cruz Biotechnology	CAT#sc-393325; RRID:AB_2895257
Podocalyxin	R&D Systems	CAT#AF1556; RRID:AB_354858
Decorin	R&D Systems	CAT#AF1060; RRID:AB_2090386
Osteocalcin	Abcam	CAT#ab93876; RRID:AB_10675660
Osteocalcin	ThermoFisher Scientific	CAT# PA5-78870; RRID:AB_2745986
VCAM-1	R&D Systems	CAT#AF643; RRID:AB_355499
E-Cadherin	R&D Systems	CAT#AF748; RRID:AB_355568
FSP1	Millipore	CAT#07-2274; RRID:AB_10807552
NG2	Millipore	CAT#AB5320; RRID:AB_91789
ICAM-1	R&D Systems	CAT#AF796; RRID:AB_2248703
ICAM-1	Abcam	CAT#ab53013; RRID:AB_870702
Fibronectin	Abcam	CAT#ab2413; RRID:AB_2262874
CXCR4	Abcam	CAT#ab124824; RRID:AB_10975635
CD150 (TC15-12F12.2)	BioLegend	CAT#115903; RRID:AB_313682
CD48 (HM48-1)	BD Pharmingen	CAT#561242; RRID:AB_10644381
Sca1 (E13–161.7)	BioLegend	CAT#122506; RRID:AB_756191
c-kit (2B8)	BioLegend	CAT#105814; RRID:AB_313223
TER-119	TOBO bioscience	CAT#305921; RRID:AB_2621651
Gr1	BioLegend	CAT#108411; RRID:AB_313376
CD2 (RM2-5)	BioLegend	CAT#100112; RRID:AB_2563090
CD3	BioLegend	CAT#100236; RRID:AB_2561456
CD5	Miltenyi Biotec	CAT#130-102-482; RRID:AB_2658610
CD8	TOBO bioscience	CAT#50-0081; RRID:AB_2621741
CD16/32	BD Pharmingen	CAT#553142; RRID:AB_394657
VEGFR3	Life Technologies	CAT#13-5988-82; RRID:AB_466851
CD45-FITC	TONBO Biosciences	CAT#35-0451-U025; RRID:AB_2621689
Isotope control (IgG1k-PE)	Life Technologies	CAT#12-4301-82; RRID:AB_470047
Isotope control (IgG2a kappa)	Life Technologies	CAT#12-4321-82; RRID:AB_470052
IgG-AF647	ThermoFisher Scientific	CAT#A-21447; RRID:AB_2535864
Mac-1	BioLegend	CAT#101204; RRID:AB_312787
B220	Miltenyi Biotec	CAT#130-101-928; RRID:AB_2660454
LYVE1-PE	Life Technologies	CAT#12-0443-82; RRID:AB_2802179
Goat anti-Rabbit IgG (H + L) Alexa Fluor 546	ThermoFisher Scientific	CAT#A-11035; RRID:AB_2534093
Donkey anti-Goat IgG (H + L) Alexa Fluor 488	ThermoFisher Scientific	CAT#A-11055; RRID:AB_2534102
Donkey anti-Goat IgG (H + L) Alexa Fluor 647	ThermoFisher Scientific	CAT#A-21447; RRID:AB_2535864
Donkey anti-Goat IgG (H + L) Alexa Fluor 546	ThermoFisher Scientific	CAT#A-11056; RRID:AB_2534103
Donkey anti-Rat IgG (H + L) Alexa Fluor 488	ThermoFisher Scientific	CAT#A-21208; RRID:AB_2535794
Donkey anti-Rabbit IgG (H + L) Alexa Fluor 488	ThermoFisher Scientific	CAT#A-21206; RRID:AB_2535792
Donkey anti-Rabbit IgG (H + L) Alexa Fluor 647	ThermoFisher Scientific	CAT#A-31573; RRID:AB_2536183
Donkey anti-Rat IgG H&L (Alexa Fluor 647)	Abcam	CAT#ab150155; RRID:AB_2813835
Donkey anti-Rat IgG H + L (Alexa Fluor 594)	ThermoFisher Scientific	CAT#A-21209; RRID:AB_2535795
Streptavidin Alexa Fluor 647 conjugate	ThermoFisher Scientific	CAT#S21374; RRID:AB_2336066

**Biological samples**		

Human Femoral Condyle Tibial Plateau (male and female donors)	Oxford Musculoskeletal BioBank (OMB)	OMB: 0131 - 0488

**Chemicals, peptides, and recombinant proteins**		

Tamoxifen	Sigma-Aldrich	CAT#T5648
SAR131675	Selleckchem	CAT#S2842
Ethanol	Sigma-Aldrich	CAT#1070172511
Glutaraldehyde	Sigma-Aldrich	CAT#340855
H_2_O_2_	Sigma-Aldrich	CAT#H1009
Paraformaldehyde	Sigma-Aldrich	CAT#P6148
PBS (10X)	VWR	CAT#437117K
EDTA	Sigma	CAT#E6758
Calcein	Sigma	CAT#C0875
Urea	VWR	CAT#28876.367
Glycerol	VWR	CAT#24388.260
Triton X-100	Sigma-Aldrich	CAT#T8787
Collagenase A	Merck	CAT#10103578001
Collagenase IV	Gibco	CAT#17104-019
Dispase II, powder	ThermoFisher Scientific	CAT# 17105041
DNase I	Sigma-Aldrich	CAT# D4527-20KU
Fetal Bovine Serum	Sigma-Aldrich	CAT#F7524
Calf serum	Sigma-Aldrich	CAT#C8056
Donkey serum	Abcam	CAT#ab7475
Penicillin/Streptomycin	Lonza	CAT#DE17-602E
Amphotericin	Sigma-Aldrich	CAT#A2942
DMSO	Sigma-Aldrich	CAT#D5879
Ethyl cinnamate	Sigma-Aldrich	CAT#112372
Polyethylene glycol	Sigma	CAT#447943
Evans Blue	Sigma-Aldrich	CAT#E2129
Sucrose	Sigma-Aldrich	CAT#S9378
Gelatin	Sigma-Aldrich	CAT#G2625
Polyvinylpyrrolidone	Sigma-Aldrich	CAT#PVP360
O.C.T	VWR	CAT#361603E
DAPI	Sigma-Aldrich	CAT#D9542
Fluoromount-G	Invitrogen	CAT#00-4958-02
TOPRO-3	Invitrogen	CAT#T3605
Tris	Sigma	CAT#T1503
NaCl	Sigma	CAT#S7653
EGTA	Sigma-Aldrich	CAT#E4378
Sodium deoxycholate	Sigma-Aldrich	CAT#D6750
Methanol	ThermoFisher Scientific	CAT#M/4058/17
5-Fluorouracil (5-FU)	Merck Life Science	CAT#F6627
Dynabeads	Invitrogen	CAT#11035
Dynabeads	Invitrogen	CAT#11203D
SDF1 protein	Abcam	CAT#ab270067
CXCL12/SDF-1 alpha Protein	R&D Systems	CAT#460-SD
IL-6 Protein	R&D Systems	CAT#406-ML
Ammonium chloride	Sigma-Aldrich	CAT#213330
Potassium	Merck	CAT#244864

**Critical commercial assays**		

Prox1 ELISA kit	Abbexa	CAT#abx382475
Prox1 ELISA kit	Abbexa	CAT#abx390312
IL6 ELISA kit	Biotechne	CAT#M6000B
CXCL12 ELISA kit	Biotechne	CAT#MCX120
TaqMan Gene Expression Master Mix	Applied Biosystems	CAT#4369510
Super-Script IV First-Strand Synthesis System	Invitrogen	CAT#18091200
PowerUp SYBR Green Real-Time PCR Master Mix	Applied Biosystems	CAT#A25742
High-Capacity cDNA Reverse Transcription Kit	Applied Biosystems	CAT#4368814
Zombie violet live/dead dye	Biolegend	CAT#423113
iScript™ cDNA Synthesis Kit	Bio-Rad	CAT#1708890
RNeasy Micro Kit	QIAGEN	CAT#74004
RNeasy Mini Kit	QIAGEN	CAT#74104
RNeasy Plus Micro Kit	QIAGEN	CAT#74034
CD45 cell depletion kit	Invitrogen	CAT#8804-6864-74

**Experimental models: Organisms/strains**		

Mouse: C57BL/6J	Charles river	Strain#632
Mouse: Myh11-cre/ER^T2^	The Jackson Laboratory	Strain#019079; RRID:IMSR_JAX:019,079
Mouse: IL-6^-^	The Jackson Laboratory	Strain#002650; RRID:IMSR_JAX:002,650
Mouse: Prox1-Cre-ER^T2^	The Jackson Laboratory	Strain#022075; RRID:IMSR_JAX:022,075
Mouse: Cxcl12^tm1.1Sjm^/J	The Jackson Laboratory	Strain#022457; RRID:IMSR_JAX: 022,457
Mouse: Prox1-tdTomato	The Jackson Laboratory	Strain#018128; RRID:IMSR_JAX:022,457
Mouse: ROSA26^eGFP−DTA^	The Jackson Laboratory	Strain#006331; RRID:IMSR_JAX:006,331
Mouse: SvJae-Pdgfrb^tm11Sor^/J	The Jackson Laboratory	Strain#010977; RRID:IMSR_JAX:010,977
Mouse: Gli1^tm3(cre/ERT2)Alj^/J	The Jackson Laboratory	Strain#007913; RRID:IMSR_JAX:007,913
Mouse: Cspg4-cre/Esr1^∗^	The Jackson Laboratory	Strain#008538; RRID:IMSR_JAX:008,538
Mouse: Cxcl12^tm2.1Sjm^/J	The Jackson Laboratory	Strain#022458; RRID:IMSR_JAX:022,458
Mouse: Adipoq-Cre/ER^T2^	The Jackson Laboratory	Strain#025124; RRID:IMSR_JAX:025,124
Mouse: mT/mG	The Jackson Laboratory	Strain#007576; RRID:IMSR_JAX:007,576
Mouse: Lyve-1 Cre^+^	The Jackson Laboratory	Strain#012601; RRID:IMSR_JAX:012,601
Mouse: Ai9(RCL-tdT)	The Jackson Laboratory	Strain#007905; RRID:IMSR_JAX:007,905

**Software and algorithms**		

GraphPad Prism (version 7)	GraphPad Software, La Jolla, CA, USA	https://www.graphpad.com
LaVision BioTech ImSpector	MACs, Miltec bio	N/A
Imaris (version 9.6.0)	Bitplane	https://imaris.oxinst.com/packages
Imaris File converter (version 9.6.1)	Bitplane	https://imaris.oxinst.com/packages
Zen Black (version 3.1)	Zeiss (Jena, Germany)	https://www.zeiss.com/microscopy/int/products/microscope-software/zen-lite
FlowJo software (version 10.7.2)	BD	https://www.flowjo.com/solutions/flowjo/downloads
FACSDiva software	BD Bioscience	https://www.bdbiosciences.com/en-us/instruments/research-instruments/research-software/flow-cytometry-acquisition/facsdiva-software
Adobe Photoshop CC	Adobe	https://www.adobe.com/uk/products/photoshop
Adobe Illustrator CC	Adobe	https://www.adobe.com/uk/products/illustrator

**Others**		

Low-profile blades	Leica	CAT#14035838382
Miltenyi-LaVision Biotec Ultra-Microscope II	Miltenyi Biotec	N/A
Zeiss Laser scanning confocal microscope LSM880	Carl Zeisis AG	N/A
Water bath	Stuart	CAT#SBS40
Gamma Cell Irradiator	Nordion	N/A
BD LSR II Flow Cytometer	BD Biosciences	N/A
BD LSR Fortessa X-20	BD Biosciences	N/A
Superglue	No-Nonsense	CAT#26780
Microscopic Slides	ThermoFisher Scientific	CAT#1237-3118
Disposable Base molds	Ted Pella. INC	CAT#27147-2
Disposable Base molds	Ted Pella. INC	CAT#27147-4
Cover glass	VWR	CAT#631-0135
Corn oil	Merck	CAT#PHR2897

### Resource availability

#### Lead contact

Further information and request for resources and reagents should be directed to and will be fulfilled by the lead contact, Anjali Kusumbe (anjali.kusumbe@rdm.ox.ac.uk).

#### Materials availability

All materials generated in this study are available from the lead contact upon request.

### Experimental models and subject details

#### Mice

C57BL/6 mice (Charles River) were used as wild type mice for all analysis unless stated otherwise. Juvenile mice were aged between 3 and 6 weeks, adult mice between 8 and 12 weeks, and aged mice were >55 weeks. Both male and female mice were used. Details of the transgenic mouse lines are listed in Key resources table. In drug treatments, mice were randomly allocated for treatment and littermates were used as sham.

For SAR131675 treatment, C57BL/6 mice received SAR131675 (Selleckchem) with a dose of 100 mg/kg via the intraperitoneal route. In this experiment sham mice were injected with PBS (PBS).

All animals were maintained following Principles of Laboratory Animal Care formulated by the National Society for Medical Research and the Guide for the Care and Use of Laboratory Animals (National Academies Press, 2011). According to institutional guidelines and laws, all experiments were performed following the protocols approved by the local University of Oxford and Imperial College London Animal Welfare and Ethical Review Board and the UK Government Home Office (Animals Scientific Procedures Group).

#### Human samples

Bone marrow biopsies were taken from the patients (male and female; age range: 61-77 years) pathologically diagnosed with lung cancer in Dazhou Central Hospital from June 2020 to January 2021. Iliac or pyramidal cancellous samples were collected by a professional orthopedic surgeon in the interventional room. The study was approved by the ethics committee, and informed consent of patients was obtained (IRB2020023). Tissue samples were obtained from the Oxford Musculoskeletal Biobank and were collected with informed donor consent in full compliance with national and institutional ethical requirements, the UK Human Tissue Act, and the Declaration of Helsinki (HTA Licence 12,217 and Oxford REC C 09/H0606/11). The research was supported by the National Institute for Health Research (NIHR) Oxford Biomedical Research Center (BRC). Disclaimer: The views expressed are those of the author(s) and not necessarily those of the NHS, the NIHR or the Department of Health. Male and female donors with age range 56-87 years were involved in the study.

### Method details

#### Tamoxifen treatment for inducible gene deletion and genetic lineage tracing

Genetic deletion and lineage tracing was performed as previously described.^12^ Briefly, tamoxifen (Sigma-Aldrich, T5648) was freshly prepared by first dissolving in 100% ethanol and then suspended in corn oil to a final concentration of 5 mg/mL. For tamoxifen-induced genetic lineage tracing, Cre ER^T2^ mouse lines, as indicated in the figure legends, were used. To induce Cre activity, tamoxifen was administered orally at a dose of 50 mg/kg for three consecutive days. In all the experiments in this study tamoxifen injections were performed in both Cre^+^ and Cre^−^ mice as in previous studies.^1^^,^^2^^,^^12^^,^^84^ At the indicated time, mice were euthanized by CO_2_ asphyxiation, and tissues were collected for analysis.

#### Bone collection, fixation, and decalcification for light sheet imaging

Freshly dissected bones were washed with PBS, (PBS, VWR, 437117K) and then quickly moved to an ice-cold fixative solution comprising 4% (w/v) paraformaldehyde, (PFA, Sigma-Aldrich, P6148) and 0.05% (v/v) glutaraldehyde (Sigma-Aldrich, 340,855) for 2.5 h. The fixative solution should be freshly made and should be ice-cold during use. Before the fixation step, the adjoining muscles and fat attached to the bone should be entirely removed by scraping as their presence interfere with generating whole bone overviews during imaging. An important step in the method is to remove the adjacent soft tissues and fat by scraping. This not only enables rapid clearing but most importantly provide accurate bone structural features for imaging. The soft tissue removal process with scraping and the collagenase digestion impacts periosteal cells. Thus, the method is suitable for analysis of bone marrow and cortical bones but not the periosteum.

The bone samples were then washed with PBS three times at room temperature on a rocker platform for 5 min for each washing step. The fixed bones can be stored for up to four days at 4°C before going forward with the decalcification. For decalcification, skeletal tissues were treated with 0.5 M EDTA solution at a pH of 7.4 and incubated at 4°C for 24 h under constant rotation. After decalcification, the samples were washed three times with PBS on a rocker (5 min at each wash).

Bones were submersed in an increasing ethanol gradient of 50%, 80%, and 100% for 30 min each for dehydration. 100% ethanol was changed twice after every 20 min. These dehydration buffers should be used ice-cold. Samples were then immersed in 5% (v/v) H_2_O_2_ for 2 h (Sigma-Aldrich, H1009) for bleaching. Tissues were then rehydrated with the decreasing ethanol gradient followed by washing three times with PBS for 20 min.

#### Immunolabeling of whole bones

The samples, after fixation and bleaching, were subjected to antigen retrieval and permeabilization with an ice-cold solution containing 25% (w/w) Urea (VWR, 28,876.367), 15% (w/w) Glycerol (VWR, 24,388.260), 15% (w/w) Triton X-100 (Sigma-Aldrich, T8787), and double distilled water 45% (w/v) incubated for 5 h at 4°C. Samples can then be subjected to enzyme-based matrix digestion with 0.2% (w/v) Collagenase (Merck, 10,103,578,001) in PBS at 37°C for 30 min on constant shaking. The samples were then washed twice for 5 min with the wash buffer made of 2% (v/v) FBS (Sigma-Aldrich, F7524) in PBS on a rocker platform.

The samples were moved to a blocking solution (freshly prepared) containing 10% (v/v) donkey serum (Abcam, ab7475), 10% (v/v) DMSO (Sigma-Aldrich, D5879), and 0.5% (v/v) Triton X-100 in PBS, at 37°C for 20 min. After blocking, the tissues were then incubated with the Alexa fluor conjugated antibodies or with the unconjugated primary antibodies as per the need. Primary antibody (antibodies are listed in the Key resources table) solution was prepared in antibody dilution buffer containing 2% (v/v) donkey serum, 10% (v/v) DMSO, and 0.5% (v/v) Triton X-100 in PBS. The tissues were kept overnight or 14-16 h at 37°C in a water bath (Stuart, SBS40), shaking at 70 rpm after the incubation period, samples were washed with a solution composed of 2% (v/v) donkey serum and 0.5% (v/v) Triton X-100 in PBS for 3 h at 37°C in the water bath shaking at 70 rpm. The solution was changed every 15 min for the first hour and then after every 30 min during the washing. In the case of staining with unconjugated primary antibodies, after washing, the samples were incubated with dye conjugated secondary antibodies for 6-8 h at 37°C in a water bath shaking at 70 rpm. The secondary antibody is diluted with antibody dilution buffer. Following incubation, the samples were washed as per the protocol described for the primary antibody.

#### Dehydration and clearing of bones

After immunostaining, the tissues were dehydrated in an increasing gradient of 30%, 50%, and 80% of ethanol for 30 min each under gentle rotation at room temperature. The samples were then immersed in 100% methanol for 1 h with ethanol changes after every 20 min during this duration. The methanol was then completely removed, and the samples were rinsed twice with Ethyl cinnamate (ECi) (Sigma-Aldrich, 112,372) for 5 min each. Bones were then cleared with a clearing solution containing 80% (v/v) ECi and 20% (v/v) polyethylene glycol (PEG) (Sigma, 447,943) under gentle rotation at room temperature for 30-60 min.

#### Evans blue

We used a direct lymphatic visualization procedure to analyze murine lymphatic vessels in bone. Evans blue, a vital dye, binds with high-affinity tissue protein and is selectively and exclusively absorbed from interstitial space by initial lymphatic vessels.^12^^,^^43^^,^^44^^,^^45^ Evans Blue (Sigma Aldrich, E2129) was injected subcutaneously into the inner leg, medial to the tail, and footpad.^12^^,^^41^^,^^43^^,^^46^^,^^47^ The injection site was blebbed slightly before lymphatic vessels gradually take up the Evans blue. The dye was allowed to travel through lymphatics and accumulate in tibial bones and evaluated 3-6 h post-injection. Following that, mice were sacrificed, and bones were dissected and immediately placed in ice-cold 2% (v/v) PFA for 4 h. Cryosectioning was performed as previously described.^10^^,^^12^^,^^41^^,^^43^^,^^46^^,^^47^ To image Evans blue in the thick cryosections, the 633 nm laser wavelength was used.

#### Irradiation

C57BL/6J mice were whole-body irradiated with two doses of 1080 rad (Gammacell irradiator) at least 2 h apart. One million bone marrow cells from wild type or mT/mG mice were injected into the tail vein of anesthetized mice. The recipient mice were maintained on antibiotic water for 14 days after transplantation and then switched to standard water.

#### Long-term competitive reconstitution assay

Cells were transplanted intravenously into the tail vein of anesthetized mice. For competitive reconstitution assays, 3×10^5^ donor bone marrow cells along with 3×10^5^ recipient bone marrow cells were transplanted. For secondary transplantation assays, 1x10^6^ bone marrow cells from primary recipients and 1x10^6^ compromised bone marrow cells which are the previously transplanted-recipient-type, were transplanted. Mice were maintained with antibiotic water for 14 days and then shifted to standard water. Recipient mice were regularly bled to assess the level of donor-derived blood cells, including B cells, T cells and myeloid cells. Blood was subjected to ammonium chloride/potassium-based red cell lysis before antibody staining. Antibodies including anti-CD45.2 (104, 1:100), anti-CD45.1 (A20, 1:100), anti-Gr1 (8C5, 1:800), anti-Mac-1 (M1/70, 1:400), anti-B220 (6B2, 1:800), and anti-CD3 (KT31.1, 1:100) were used to stain the cells. Cells were then analyzed by flow cytometry. All antibodies were obtained from eBioscience (San Diego, CA) or BD Bioscience (San Jose, CA).

#### Flow cytometric analysis of hematopoietic cells

Cells from the bone marrow were isolated by crushing the femurs with a mortar and pestle in a DMEM cell culture medium supplemented with 2% heat-inactivated bovine serum. The cells were dissociated to a single-cell suspension by passing within a 25G needle and then filtering through a 40 μm cell strainer. These antibodies were used for staining HSCs: CD150 (TC15-12F12.2, 1:150), CD48 (HM48-1, 1:150), Sca1 (E13–161.7, 1:150) and c-kit (2B8, 1:150). Following antibodies were used against lineage markers: TER-119 (1:150), B220 (1:150), anti-Gr1 (1:150), CD2 (RM2-5, 1:150), CD3 (1:150), CD5 (53–7.3, 1:200) and CD8 (53–6.7, 1:200). Unless indicated, antibodies for flow cytometry were purchased from eBioscience (San Diego, CA) or BD Bioscience (San Jose, CA). Samples were analyzed using FACS BD Fortessa X-20 or BD LSRII flow cytometers (BD Biosciences, San Jose, CA). Data were analyzed on FACSDiva software (BD Biosciences, San Jose, CA).

#### Flow cytometric analysis of lymphatic endothelial cells

For flow cytometry of lymphatic endothelial cells (LECs), one tibia bone was collected and thoroughly cleaned to remove the attached muscle. The bone was then crushed using a pestle and mortar, followed by incubation in a collagenase A solution (0.8 mg/mL) at 37°C for 1 h. Isolated cells were then washed and passed through a 100 μm filter prior to subsequent staining with a zombie violet live/dead dye (Biolegend, 423,113, 1:500) at room temperature for 20 min in accordance with manufacturers’ instructions. Cells were then washed in buffer (5mM EDTA (1.46 g/L), 2% FBS - Fetal Bovine Serum) and immunostained with primary antibodies, CD16/32 (BD Pharmingen, 553,142, 2.4G2, 1:100) and Podoplanin (R&D systems, AF3244, polyclonal, 1:200) or their isotype control antibody (Life Technologies, 13-4321-82, IgG2a kappa, 1:200 or R&D Systems, AB-108-C, IgG, 0.2:200, respectively) for 45 min at 4°C. After further washing, cells were subsequently incubated with CD45-FITC (TONBO Biosciences, 35-0451-U025, 30-F11, 1:500) and LYVE1-PE (Life Technologies, 12-0443-82, ALY7, 1:200) or it’s isotype control antibody (Life Technologies, 12-4301-82, IgG1k-PE, 1:200) and Streptavidin-AF647 (Life Technologies, S21374, 1:500) or IgG-AF647 (ThermoFisher, A-21447, 1:500) for 45 min at 4°C. After washing, data was acquired on BD Fortessa X-20 or BD LSRII flow cytometers and analyzed using FlowJo software (version 10.7.2, BD).

#### Sample preparation for immunohistochemistry

Bones were dissected and placed in ice-cold 2% paraformaldehyde (PFA) in PBS and kept on ice for 4 h. Bones were then processed as previously described.^10^ Briefly, following washes in PBS, bone samples were placed in 0.5M EDTA (pH 7.4) for at least 36 h, dehydrated in 20% sucrose (Sigma-Aldrich, S9378), and 2% polyvinylpyrrolidone (PVP, Sigma-Aldrich, PVP360) for 48 h. Bones were then embedded in 20% sucrose (Sigma-Aldrich, S9378), 2% polyvinylpyrrolidone (PVP, Sigma-Aldrich, PVP360) and 8% gelatin (Sigma-Aldrich, G2625). Samples were sectioned at 100 μm thickness by Leica CM3050 cryostat with low-profile blades (Leica, 14,035,838,382) and air-dried before placing them in the freezer for storage.

#### Immunostaining on thick bone slices

For immunostaining, bone sections were air-dried for 15 min and rehydrated for 5 min in PBS. Sections were permeabilized in 0.3% Triton X-100 for 10 min and blocked in 5% donkey serum at room temperature (RT). Samples were incubated with the primary antibodies. Primary antibodies were diluted in blocking buffer overnight at 4°C. A list of primary antibodies are provided in the Key resources table. Samples were washed several times at RT for 5 min each in PBS and incubated with Alexa fluor conjugated secondary antibodies listed in the Key resources table and with the nuclear marker TOPRO-3 or 4′,6-Diamidino-2-Phenylindole (DAPI) (1:1000) for 2 h at RT. Following several washes in PBS, samples were mounted with glass coverslips using Fluoromount-G (Invitrogen, 00-4958-02). Staining without primary antibodies and only secondary antibodies were used as negative controls.

#### Light sheet imaging set-up and image acquisition

The cleared samples were imaged on the Miltenyi-LaVision Biotech Ultra-Microscope II with the LaVision BioTech ImSpector software (MACs, Miltec bio). The microscope was equipped with a 2X objective lens for the zoom body with a manual zoom of 0.63X–6.3X. The objective was fitted with a Dipping Cap [5.7 mm], including correction Optics for Olympus MVPLAPO 2X. The microscope was fitted with the 405-100, 488-85, 561-100, 639-70 and 785-75 laser lines and the images were captured with the *Neo* sCMOS camera (Andor). For image acquisition, cleared samples were manually attached with a drop of Superglue (No-Nonsense, UK) to the sample holder adapter. Once the sample was firmly affixed to the sample holder, they were gently immersed in ECi in a quartz glass cuvette and excited with light sheets (30-90 mm, dependent on organ size) of different wavelengths (405, 488, 561, 640 and 785 nm). The individual TIFF raw data images were converted by Imaris File converter (version 9.6.1, Bitplane) and then analyzed by Imaris software (version 9.6.0, Bitplane).

#### Confocal imaging set-up and image acquisition

Z-stacks of immunostained sections were imaged on the Zeiss Laser scanning confocal microscope LSM880 using the 20X Plan Apo/0.8 dry lens and 10X Plan Apo 0.45 WD = 2.0 M27 dry lens. The imaging set-up consisted of a Zeiss laser scanning microscope 880 equipped with Axio Examiner, laser lines: 405, 453, 488, 514, 561, 594, and 633 nm, Colibri 7 epifluorescence light source with LED (light-emitting diode) illumination, four objectives, fast scanning stage with PIEZO XY, 32-channel gallium arsenide phosphide detector (GaAsP) PMT (photomultiplier tube) plus two-channel standard PMT, acquisition, and analysis software including measurement, multichannel, panorama, extended manual focus, image analysis, time-lapse, z stack, extended focus, autofocus, and with additional modules: Experiment Designer and Tiles and Position.

Large regions through the thick sections were imaged using the tile scan function, and images were stitched with a 10% overlap using Zen Black (version 3.1, Zeiss) software. In order to visualize the boundary of the organ, the autofluorescence from the 405 channel was converted into greyscale, and the 30% opaque image was manually overlayed with the corresponding TIFF file generated from Imaris. Imaris, Adobe Photoshop and Adobe Illustrator software were used to generate, analyze and compile images.

#### Image analysis and quantifications

Slices/Z-stacks of images acquired on the light sheet and confocal microscope were processed and reconstructed in three dimensions with Imaris software (version 9.6.0). Imaris, Adobe Photoshop, and Adobe Illustrator software were used for image processing and analysis in line with the journal’s guidance for image processing.

Maximum intensity projections were analyzed using Imaris (version 9.6.0). Vessel width was calculated using the distance tool in Imaris on single slices. Quantification of cell numbers was done on a z stack of images in Imaris using the automatic spot detection feature and manually annotated to remove any signal that was determined to be non-specific. For the analysis of lymphatic vessel density, the Imaris Surface Analysis XTensions tool was used. Briefly, using the Crop 3D tool, the total tissue volume was acquired via the Volume Statistics function in Imaris. Then, a single channel of a lymphatic endothelial marker was reconstructed in 3D using the Surface function, and the tissue volume of lymphatic vessels was measured using the Surface Statistics function. The lymphatic vessel density was calculated by dividing the tissue volume of lymphatic vessels in the numerator by the total tissue volume of the whole organ in the denominator.

#### 3D surface reconstruction

3D surface rendering in images was applied using the surface module in Imaris. Briefly, the ROI was defined, and a single channel of lymphatic or blood vessels, perivascular cells, or matrix markers was reconstructed with surface segmentation. Followed by the smoothness of the ROI, the background subtraction option was used for the threshold settings. After the threshold adjustment, the manual option was set up to reach a proper value according to the preview. Last, the resulting images were visually inspected to manually remove small individual segmented components of high sphericity, which were regarded as noise.

#### RNA isolation for outer surface of bones and cortical bones

Freshly dissected bones were prepared for light sheet imaging as described above by removing adjacent fat and scraping the cells around the outer surface of the bones. To isolate the RNA from the outer surface of bones, intact femurs were dipped in the RNA lysis buffer (RNeasy Plus Micro Kit, QIAGEN) for 20 min. After this the outer surface of bones in lysis buffer were scraped with a scalpel blade. To isolate the RNA from the cortical bones, femurs were cut from the top and bottom and then flushed with PBS using a syringe to remove the bone marrow. The flushed bones (cortical bones) were then crushed with mortar and pestle. Crushed bones were then subjected to digestion with 0.2% collagenase IV, dispase (1.25 U/ml) (ThermoFisher Scientific, 171,055-041), and DNase I (7.5 mg/mL) (Sigma-Aldrich, D4527-10KU) for 45 min at 37°C to prepare single-cell suspension for the isolation of RNA. RNA isolation was performed RNeasy Plus Micro Kit (QIAGEN, 74,034) according to the manufacturer’s instructions.

#### RNA isolation from whole bones

Total RNA was isolated according to manufacturer’s protocol (RNeasy Mini Kit, QIAGEN). A total of 100 ng RNA per reaction was used to generate cDNA with the iScript cDNA Synthesis System (Bio-Rad). Bones were crushed with mortar and pestle.^12^ Crushed bones were then subjected to digestion with 0.2% collagenase IV, dispase (1.25 U/ml) (ThermoFisher Scientific, 171,055-041), and DNase I (7.5 mg/mL) (Sigma-Aldrich, D4527-10KU) for 45 min at 37°C for the isolation of RNA.

#### qPCR

qPCR was done using TaqMan gene expression assays on the ABI PRISM 7900HT Sequence Detection System.^1^^,^^2^^,^^12^^,^^52^^,^^85^ The FAM-conjugated TaqMan probes were used with the TaqMan Gene Expression Master Mix (Applied Biosystems, 4,369,510). Gene expression assays were normalized to endogenous VIC-conjugated Actb probes as standard. RNA samples were rapidly processed for cDNA preparation using the Super-Script IV First-Strand Synthesis System (Invitrogen, 18,091,200). To conduct qPCR, FAM-conjugated TaqMan probes were applied with TaqMan Gene Expression Master Mix (Applied Biosystems, 4,369,510).

#### Micro-CT analysis and histomorphometry

Tibiae were collected from mutants and their littermate controls; the attached soft tissue in the bone was removed thoroughly and fixed in 2% paraformaldehyde. The fixed tibiae were analyzed using micro-CT (μCT 100). Following scan settings were used Voxel size 6.0 μm, FOV 10.236 mm, Image matrix 1706 × 1706 × 550, Slices 550, Scanned region 3.3 mm, X-ray voltage 70 kVp, and Intensity 85 μA, 6 W.

Calcein double labeling was performed to calculate Bone Formation Rate (BFR) and mineral apposition rate (MAR). Mice were given intraperitoneal injections of 10 mg/kg calcein (Sigma, C0875) dissolved in 2% sodium bicarbonate solution on the 10^th^ day and third day before euthanasia. Bones were fixed in 4% PFA, embedded in 8% gelatin and 2% PVP and cryosectioned. Single plane images were acquired from the sections. Sections were stained with von Kossa method to assess mineralized bone. Representative images show cortical bone (diaphysis about 3 mm proximally from the growth plate). Mineral Apposition Rates were calculated from both cortical and trabecular bones.

#### Cartilage explant culture

Cartilage explants (0.125 g) were cultured in DMEM with 25 mmol of HEPES, penicillin/streptomycin, and amphotericin. Isolated chondrocytes were incubated in medium supplemented with 10% Fetal Calf Serum. Freeze-thawed cartilage was frozen in liquid nitrogen for 2 min and thawed in a water bath at 37°C for 3 times, after which the explants were washed thoroughly and further incubated as required.

#### Isolation of LECs for qPCR

Isolation of LECs from young and aged mouse bones. Femurs and tibiae were collected, crushed in sterile condition, digested with collagenase A (Sigma-Aldrich) at 37°C for 45 min, and passed through a 40-μm filter to obtain single-cell suspensions. CD45 positive cells were depleted from the single cell suspension of bones using BD CD45 cell depletion kit (Invitrogen, 8804-6864-74). LYVE1 antibody raised in rabbit were used to isolate LECs by magnetic bead–based separation. Positive cells were then separated by using anti-rabbit magnetic beads (Dynabeads M−280 Sheep Anti-Rabbit, ThermoFisher Scientific) following the manufacturer’s instructions. Samples were lysed in the lysis buffer of the RNeasy Mini Kit (QIAGEN). Total RNA was isolated according to the manufacturer’s protocol. cDNA conversion was performed by using a High-Capacity cDNA Reverse Transcription Kit (Applied Biosystems). qPCR was performed using Power Up SYBR Green Real-Time PCR Master Mix (Applied Biosystems) with customized primer pairs.

#### Isolation of LECs and intratibial transplantation

Isolation of LECs from young and aged mouse bones. Femurs and tibiae were collected from young and aged mice, crushed in sterile condition, digested with collagenase A (Sigma-Aldrich) at 37°C for 45 min, and passed through a 40-μm filter to obtain single-cell suspensions. CD45 positive cells were depleted from the single cell suspension of bones using BD CD45 cell depletion kit (Invitrogen, 8804-6864-74). LYVE1 antibody raised in rabbit were used to isolate LECs by magnetic bead–based separation. Positive cells were then separated by using anti-rabbit magnetic beads (Dynabeads M−280 Sheep Anti-Rabbit, ThermoFisher Scientific) following the manufacturer’s instructions. Isolated cells were resuspended in DMEM medium. 20,000 cells were intratibially injected in aged mice and mice were subsequently subjected to radiation treatment.

#### ELISA

For bone tissue lysate preparation and cartilage explant preparation, 300 μL complete extraction buffer 100 mM Tris (Sigma, T1503), 150 mM NaCl (Sigma, S7653), 1 mM EGTA (Sigma-Aldrich, E4378), 1 mM EDTA (Sigma, E6758), 1% Triton X-100 (VWR, 306324N) and 0.1% Sodium deoxycholate (Sigma-Aldrich, D6750) supplemented with protease inhibitor cocktails was added into 5 mg tissue slices. Then, samples were homogenized with an electric homogenizer and maintained at constant agitation for 2 h at 4°C. After centrifuging for 20 min at 19,000 *g* at 4°C, the samples were placed on ice. The lysate was aliquoted and stored at −80°C. PROX1, IL6 and CXCL12 levels in mouse bones and human bone biopsies were determined by ELISA kits (Abbexa Ltd, Cambridge, UK and R&D Systems) based on the manufacturer’s instructions.

#### Sample preparation for ScRNA-seq from human bone biopsies

Bone marrow biopsies were taken from the patients pathologically diagnosed with lung cancer in Dazhou Central Hospital from June 2020 to January 2021. Iliac or pyramidal cancellous samples were collected by a professional orthopedic surgeon in the interventional room. The study was approved by the ethics committee, and informed four consent of patients was obtained (IRB2020023). The obtained samples were quickly placed into the sterile Eppendorf tubes with PBS buffer for flushing the blood. Finally, the cleaned samples were placed into a sterile Eppendorf tube with 2 mL cell preservation solution before being stored in a refrigerator at 4°C.

#### Single-cell RNA sequencing data processing

Bone samples were taken from the lung cancer patients with two replicates were sequenced in a 10x genomic chromium platform with chemistry (Single Cell 3′ v3). The samples were sequenced at BGI (https://www.bgi.com). Sequencing results were demultiplexed and converted to FASTQ format using cell ranger tool kit mkfastq. The raw data were aligned to the human reference genome (GRCh38), allowing filtering barcode and UMI count using cell ranger count (cellranger-5.0.0).

#### Cell-type clustering analysis and marker identification

The feature count matrix was further processed using Seurat (CRAN, version 4.0.1).^86^ The mitochondrial and ribosomal reads were excluded from the analysis. Additionally, only PECAM1 positive cells are used for further analysis. After filtering, a total of 2305 cells were left for the following analysis. The data were normalized, scaled, and significant variable genes were identified using SC Trans-form.^87^ Then, the dimensionality reduction technique was applied to the dataset and cell clusters were identified (resolution = 0.4). Further, the differentially expressed genes (DEG) were identified (Wilcoxon Rank-Sum test for genes with a minimum 0.5 log fold change with thresh = 0.01 between clusters and expressed in at least 25% of cells between clusters). Cell clustering was visualized using uniform manifold approximation and projection for dimension reduction (UMAP). UMAP gene expression overlays and violin plots for cell type-specific marker genes were plotted using Seurat-specific functions.

### Quantification and statistical analysis

Panels usually represent multiple independent experiments performed on different days with different mice. Sample sizes were not pre-determined based on statistical power calculations. No randomization techniques were used. Mice were allocated to experiments randomly, and samples were processed in an arbitrary order. No blinding was performed. No animals were excluded from analyzes. Variation was always indicated using SD. Data represents mean ± SEM or SD To assess the statistical significance of differences between two groups, we generally performed two-tailed unpaired Student’s t-tests. To analyze the statistical significance of differences among more than two groups, we performed one-way ANOVAs with Tukey’s multiple comparisons tests. To determine the statistical significance of differences between multiple groups when the experimental design involved multiple conditions, such as time points or cell types, in addition to differences in genotypes, we performed two-way ANOVAs with Sidak’s multiple comparisons tests. p < 0.05 was considered significant. ns: not significant, p > 0.05; ^∗^: p < 0.05; ^∗∗^: p < 0.01; ^∗∗∗^: p < 0.001; ^∗∗∗∗^: p < 0.0001. All statistical tests were performed using GraphPad with Prism7 (version 7), following its Statistics Guide. No statistical analysis was used to determine the sample size.

## Data Availability

• Data and supplementary tables and videos have been attached and are publicly available as of the date of publication. • RNA-seq data are available at E-MTAB-11560. • Any additional information required to reanalyze the data reported in this paper is available from the lead contact upon request.
